# Systematic analysis of differentially methylated expressed genes and site‐speciﬁc methylation as potential prognostic markers in head and neck cancer

**DOI:** 10.1002/jcp.28835

**Published:** 2019-05-26

**Authors:** Guohui Bai, Jukun Song, Yiwen Yuan, Zhu Chen, Yuan Tian, Xinhai Yin, Yuming Niu, Jianguo Liu

**Affiliations:** ^1^ Zunyi Medical University Zunyi Guizhou China; ^2^ Special Key Laboratory of Oral Diseases Research Stomatological Hospital Affiliated to Zunyi Medical University Guizhou China; ^3^ Department of Oral and Maxillofacial Surgery Guizhou Provincial People's Hospital Guizhou China; ^4^ Guizhou Medical Univerisity Guizhou China; ^5^ Guiyang Hospital of Stomatology Guizhou China; ^6^ Stomatology Colledge Affiliated to Zunyi Medical University Zunyi Guizhou China; ^7^ Department of Stomatology and Center for Evidence‐Based Medicine and Clinical Research, Taihe Hospital Hubei University of Medicine Shiyan China

**Keywords:** Cox regression analysis, differentially methylated genes, DNA methylation, gene set enrichment analysis, head and neck cancer, hypermethylation, hypomethylation, Kaplan–Meier survival curve, The Cancer Genome Atlas

## Abstract

Head and neck cancer (HNC) remains one of the most malignant tumors with a significantly high mortality. DNA methylation exerts a vital role in the prognosis of HNC. In this study, we try to screen abnormal differential methylation genes (DMGs) and pathways in Head–Neck Squamous Cell Carcinoma via integral bioinformatics analysis. Data of gene expression microarrays and gene methylation microarrays were obtained from the Cancer Genome Atlas database. Aberrant DMGs were identified by the R Limma package. We conducted the Cox regression analysis to select the prognostic aberrant DMGs and site‐speciﬁc methylation. Five aberrant DMGs were recognized that significantly correlated with overall survival. The prognostic model was constructed based on five DMGs (PAX9, STK33, GPR150, INSM1, and EPHX3). The five DMG models acted as prognostic biomarkers for HNC. The area under the curve based on the five DMGs predicting 5‐year survival is 0.665. Moreover, the correlation between the DMGs/site‐speciﬁc methylation and gene expression was also explored. The findings demonstrated that the five DMGs can be used as independent prognostic biomarkers for predicting the prognosis of patients with HNC. Our study might lay the groundwork for further mechanism exploration in HNC and may help identify diagnostic biomarkers for early stage HNC.

## 1 INTRODUCTION

Head and neck cancer (HNC) remains the 10th largest primary cancer in the world and the seventh leading cause of cancer death. There are approximately 400,000 oral and pharyngeal diseases, 160,000 laryngeal cancers and 300,000 deaths worldwide each year (Mehanna, Paleri, West, & Nutting, 2010; Siegel, Miller, & Jemal, 2018; Wozniak, Szyfter, Szyfter, & Florek, 2012). HNC is a heterogeneous tumor, which is characterized by lesions in the oral, pharynx, and larynx (Arantes, de Carvalho, Melendez, Carvalho, & Goloni‐Bertollo, 2014) regions. It is documented that one out of 99 people born in the United States today experienced HNC in their lifetime. It is considered an important part of the global burden of cancer (Jemal et al., 2008; Marur & Forastiere, 2008); HNC accounts for 12% of all malignancies in the world. Despite progress in treatment, HNC's 5‐year survival rate is still not favorable (Carvalho et al., 2008; Siegel et al., 2018). Early detection and risk classification of HNC is essential to improve prognosis and reduce the mortality and morbidity associated with HNC; useful diagnostic molecular biomarkers for HNC are vital for effective treatment selection.

Epigenetic changes in the development and progression of various cancers have been documented owing to rapid technological breakthroughs in whole‐genome sequencing (Kordowski et al., 2018; Molnar et al., 2018; Sweeney et al., 2009). DNA methylation usually occurs at the cytosine–phosphate–guanine (CpG) site, regulating protein function, gene expression, and RNA processing (Baylin & Jones, 2011; Portela & Esteller, 2010; Stein, 2011). Although several studies have determined that the given genes have abnormal DNA hypermethylation or hypomethylation in HNC, a combined profile and pathway of regulatory networks is still rare (Misawa et al., 2016; Misawa, Mochizuki, Endo, et al., 2017; Misawa, Mochizuki, Imai, et al., 2017; Misawa et al., 2018). In the study, the combination of gene expression and DNA methylation data was analyzed. By applying differential analysis and Cox regression analysis, five differentially methylated genes (DMGs) were identified as potential prognostic methylation genes for HNC patients. Moreover, these findings may provide new prospects for potential mechanisms based on exploring site‐specific methylation and DMGs in HNC.

## 2 MATERIALS AND METHODS

### 2.1 Data source and data processing

In the present study, RNA‐Seq data, DNA methylation data, and clinical information related to HNC patients were obtained from The Cancer Genome Atlas (TCGA) data portal (https://tcga‐data.nci.nih.gov/tcga/, August 28, 2018). The 44 adjacent nontumor samples and 502 HNC samples were included in the gene expression profiles, in which messenger RNA (mRNA) microarrays used IlluminaHiSeq RNA‐Seq arrays, while 50 adjacent nontumor samples and 530 HNC samples were included in the DNA methylation data set, in which the methylation platform used a Illumina HumanMethylation450 BeadChip.

The differentially expressed genes (DEGs) and DMGs were screened in HNC tissues compared to control tissues using the Limma/edgeR package, respectively (Ritchie et al., 2015; Robinson, McCarthy, FsSmyth, 2010). The cut‐off criteria for screening DEGs/DMGs was the false discovery rate (FDR) < 0.05 and log2 fold change) > 1.

### 2.2 Univariate cox analysis

To evaluate the impact of these genes on the HNC patient's prognosis, a univariate Cox regression analysis was used to screen the survival specific DMGs/DEGs. The prognostic genes were screened with the R package “Survival.” With the threshold of *P* values < 0.05, the DMGs/DEGs were considered as prognostic genes in the univariate Cox regression analysis. Then, common genes in DMGs/DEGs were considered as prognostic methylation genes.

### 2.3 Correlation analysis of DEGs and DMGs in HNC

The identified prognostic DMGs were selected to explore the relationship between mRNA and DNA methylation. It is widely accepted that a negative relationship between DNA methylation and mRNAs was detected. Therefore, we selected those DMGs with a negative association between DNA methylation and mRNAs, which had an *R* value in Pearson's correlation analysis < −0.3, and a *P* value < 0.05.

### 2.4 Multivariate Cox analysis

The multivariate Cox regression analysis was used to further validate these DMGs as prognosis factors. Integration of gene expression levels weighted by the regression coefficient (*β*) was used to construct a risk score model. The formula for estimating the prognosis index (PI) for each patient is as follows: (*β* × expression level of EPHX3) + (*β* × expression level of STK33) + (*β* × expression level of GPR150) + (*β* × expression level of PAX9) + (*β* × expression level of INSM1) (Bao et al., 2014; Zhang et al., 2015).

According to the threshold of the median PI, HNC patients were stratified into high‐risk and low‐risk groups. The Kaplan–Meier survival curves were plotted. To further verify that the five DMGs were also the independent indicators of other clinical factors, univariate and multivariate Cox regression analysis were performed. We used time‐dependent receiver operating characteristic (ROC) curves within 5 years to assess the prognostic performance based on the risk score model. A *P* value < 0.05 was considered statistically significant.

### 2.5 Functional enrichment analysis

We conducted differentially expressed analyses between low and high‐risk groups based on risk score models. The top 200 DEGs (100 upregulated genes and 100 downregulated genes) were screened. To better understand the biological process of the five DMGs, functional enrichment analysis was analyzed by the “ClusterProfiler” package in R software (Yu, Wang, Han, & He, 2012). The gene set enrichment analysis (GSEA) was further performed (Subramanian et al., 2005).

### 2.6 Screening of prognostic DNA methylation sites

The DNA methylation sites were downloaded through the identified prognostic five DMGs in the TCGA database. We further screened the prognostic DNA methylation sites in five DMGs through univariate Cox regression analysis and Kaplan–Meier survival analysis with the *R* “survival” package. A *P* value < 0.05 was defined as statistically significant.

### 2.7 Correlation analysis of gene expression and DNA methylation sites in HNC

The selected prognostic DNA methylation sites in five DMGs were selected to explore the relationship between mRNAs and DNA methylation sites. Meanwhile, we selected these prognostic DNA methylation sites as our object with a cut‐off threshold of the *R* value in the Pearson's correlation analysis <− 0.3 and the *P* value < 0.05. Pearson's correlation analysis was conducted with the *R* “Cor” function.

### 2.8 Survival analysis among prognostic DMGs

We selected the genes with hypermethylation/lower expression or genes with hypomethylation/higher expression in HNC. The Kaplan–Meier plot was used to explore the association between mRNA and the hypermethylation/hypomethylation genes. The log‐rank test was used to compare the survival difference between the HNC group and nontumor group in the overall survival (OS) analysis. The R “Survival” package was used to screen prognostic genes. A *P* value < 0.05 was regarded as statistically significant.

### 2.9 Construction of the nomogram model

A nomogram model was constructed in view of the results of the multivariate Cox model. To reduce overfitting bias, the nomogram was bootstrapped by 1000 resampling and quantized by the concordance index (C‐index) for verification in the TCGA HNC cohort. The C index value ranged from 0.5 to 1.0, 0.5 denoted no discrimination, and 1.0 denoted complete discrimination (Rao, 2003). The *R* “rms” packages were performed for the establishment of the model of the nomogram.

### 2.10 Validation of the DMGs with Gene Expression Omnibus (GEO) data

To validate the robustness of the hub DMGs from the TCGA data set, the DNA methylation profiles of HNC from the GEO database was searched. To determine eligible studies, we used the following search terms: “head and neck cancer” or “HNSC”. We used GEO2R online software to examine the raw data of DNA methylation and screen DMGs. GEO2R is an interactive web tool that permits users to analyze different sample sets in the GEO datasets and select differentially expressed genes under specific conditions. The adj.*P*.Val < 0.05 and |*t*| > 2 were used as the cut‐off threshold for screening DMG.

## 3 RESULTS

### 3.1 Data

The HNC data set from the TCGA data portal was downloaded. Data on RNA‐Seq, DNA methylation, and clinical profiles were collected. Demographic features are exhibited in Table 1. Two data sets were created for each clinical data set, where the rows were indexed by the TCGA patient ID and column using the following matrix: normalized count (RNA‐Seq) and beta value (DNA methylation).

**Table 1:** Demographic characteristics

Clinical variables	Clinical values (*N* = 528)
Sex (male/female)	386/142
Age (mean/std)	61.12/10.65
Race (Asian/Black/White/American India/NA)	11/48/452/3/14
Pathological stage (I/II/ III/ IV/ NA)	27/73/83/270/75
Grade (G1/G2/G3/G4/Gx/NA)	63/310/125/8/22
History of neoadjuvant treatment (Yes/No)	10/517
History of alcohol consumption (Yes/No/NA)	352/165/11
History of tobacco smoking (Lifelong nonsmoker/smokers/NA)	122/393/13
HPV status by P 16 testing (positive/negative/NA)	41/74/413
New tumor events event after initial treatment (yes/no/NA)	49/150/63
Person's neoplasm cancer status (with tumor/tumor free/NA)	140/340/48
Radiation (yes/no/NA)	126/64/338

Abbreviation: HPV, human papillomavirus.

### 3.2 Identification of aberrant DMGs in HNC

**Figure 1:**
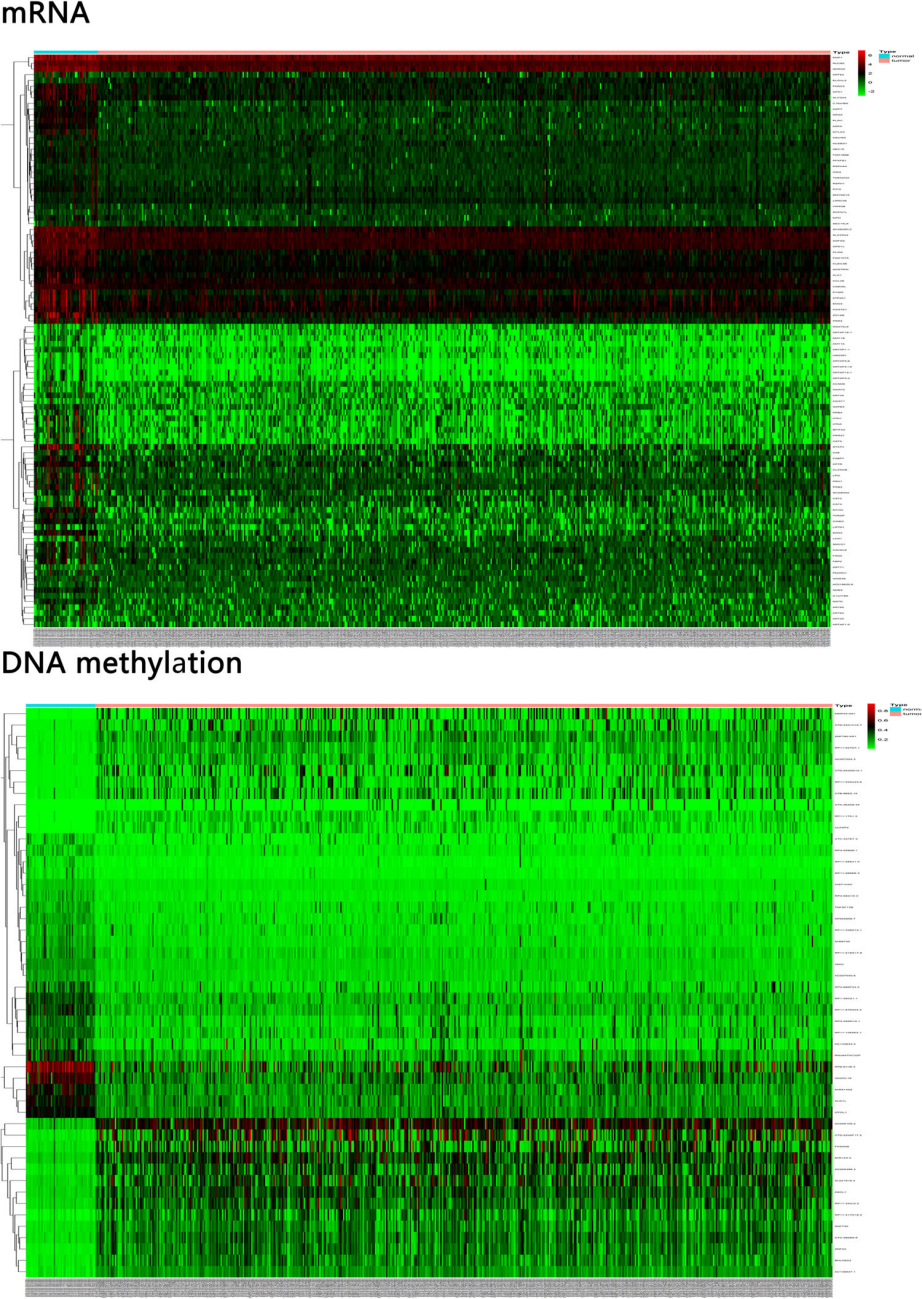
The typical heat map of DEG/DMG expression in HNC. Red, upregulation; blue, downregulation; DEGs, differentially expressed genes; DMGs, differential methylation genes [Color figure can be viewed at wileyonlinelibrary.com]

Data from the DNA methylation levels and RNA sequence were analyzed by R software to screen DMGs/DEGs, respectively. Among the DMGs in the DNA methylation profiles, 298 hypermethylation genes and 26 hypomethylation genes were identified. Of these hypermethylation genes, two DMGs yielded a > 3‐fold increased expression, which included MMP24‐AS1 and ULK4P3, and 11 DMGs exhibited over > 2‐fold increased expression. Among the hypomethylation genes, 7 DMGs exhibited over > 1.2‐fold decreased expression. For DEGs, 4,872 downregulated genes and 2,427 upregulated genes were identified. Of the overexpressed DEGs, 338 DEGs yielded a > 3‐fold increased expression, 605 DEGs exhibited over > 3‐fold decreased expression. The representative heat map of DMGs/DEGs in HNC is shown in Figure 1.

### 3.3 Univariate Cox regression of candidate DMGs

A univariate Cox regression between DMGs/DEGs and the OS in HNC patients was performed, respectively. The results show that a total of 130 DMGs/674 DEGs were significantly associated with OS with the threshold of *P* values < 0.05 (see Tables S1 and S2). The common 21 genes were screened by overlapping 129 DMGs and 674 DEGs.

### 3.4 Correlation analysis between DMG levels and DEG expression

**Figure 2:**
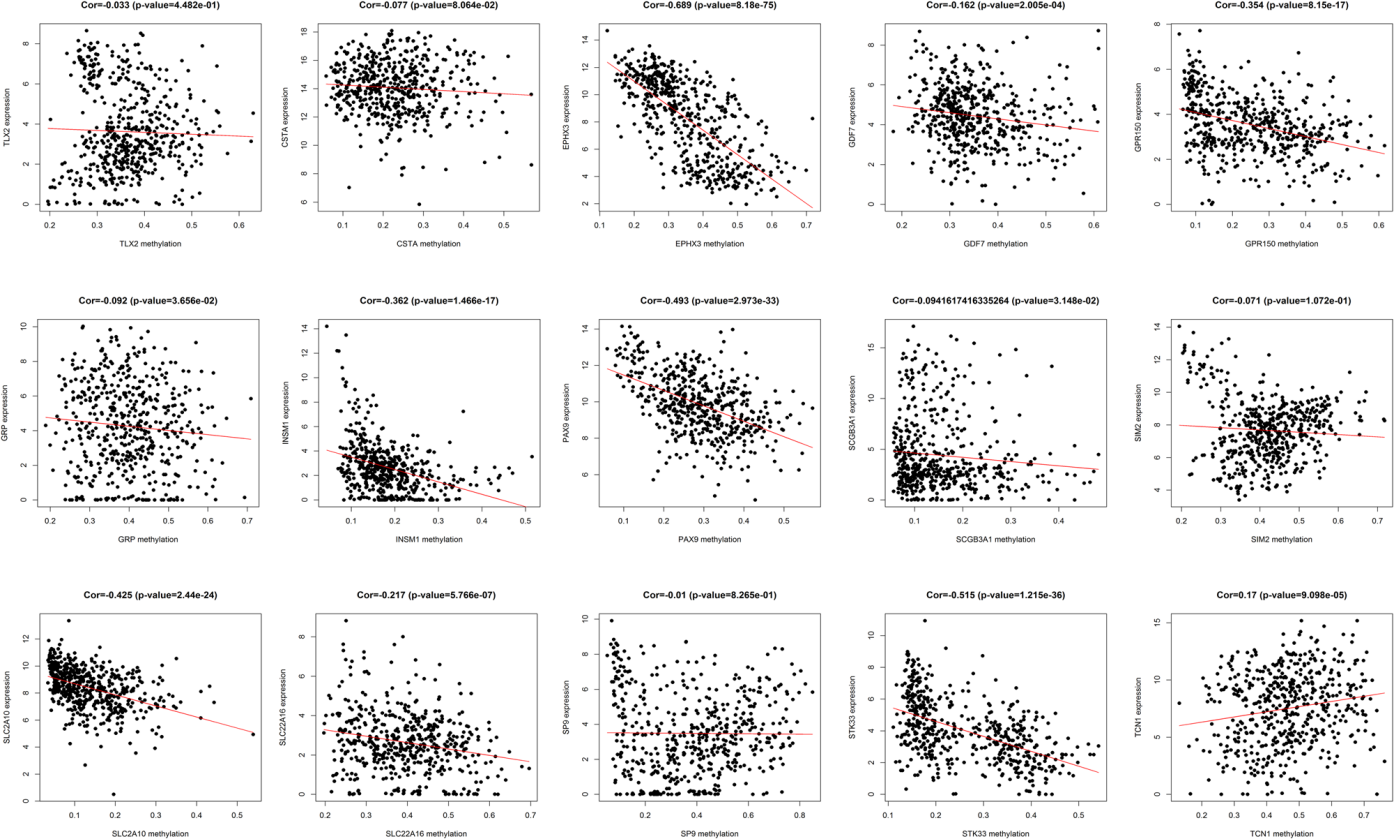
Correlation analysis between messenger RNAs and DNA methylation levels. The abscissa is the beta value of the DNA methylation gene, the ordinate is the gene expression level [Color figure can be viewed at wileyonlinelibrary.com]

The independent prognostic DMGs were used to examine the correlation between gene expression and DNA methylation. Among these 21 common DMGs, six DMGs (KRTAP3‐3, TCN1, NEUROD2, SIRPG, MMP23B, and SLITRK1) were excluded because a positive correlation was observed, 15 DMG expressions (PAX9, GDF7, STK33, TLX2, SCGB3A1, CSTA, SLC22A16, SP9, GPR150, SLC2A10, KCNC2, INSM1, SIRPG, EPHX3, SIM2, and GRP) were negatively associated with mRNA expression. Five DMGs (PAX9, STK33, GPR150, INSM1, and EPHX3) were chosen when the r‐value in the Pearson's correlation analysis was < − 0.3 (Figure 2).

### 3.5 Construction of five DMG prognostic models

**Figure 3:**
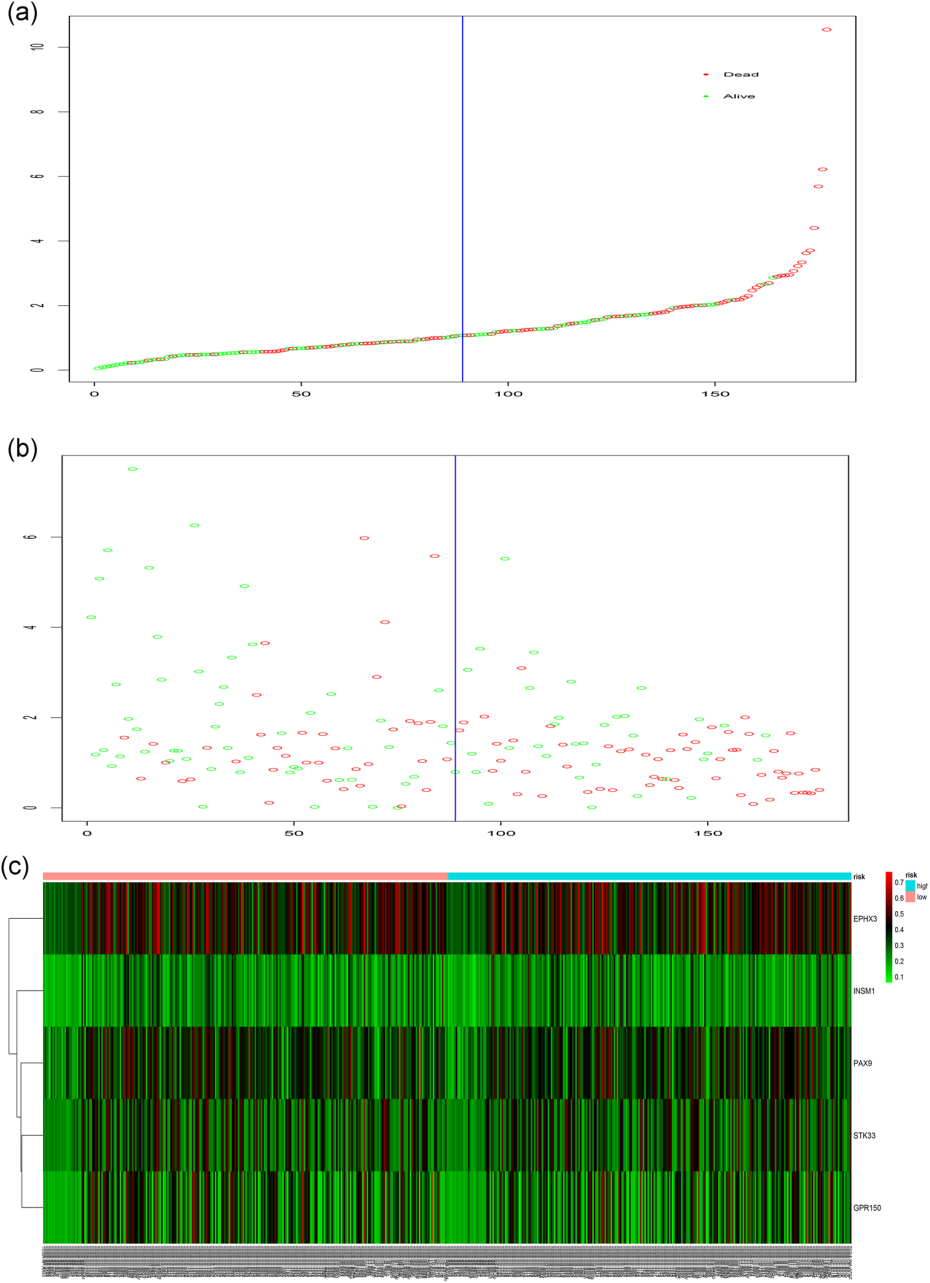
DMG risk score analysis of HNC patients in TCGA. (a) DMG risk score distribution; (b) The survival status and duration of HNC patients; (c) Heatmap of the five DMGs in HNC patients. DEGs, differentially expressed genes; HNC, head and neck cancer; TCGA, The Cancer Genome Atlas [Color figure can be viewed at wileyonlinelibrary.com]

**Figure 4:**
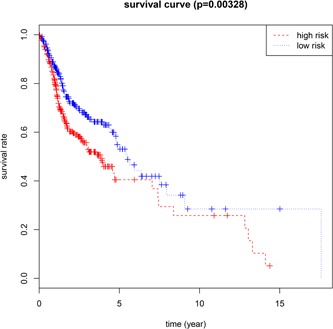
Kaplan–Meier survival analysis between the low and high‐risk groups [Color figure can be viewed at wileyonlinelibrary.com]

We performed the multivariate Cox regression analysis to validate the above results, and the five DMGs (PAX9, STK33, GPR150, INSM1, and EPHX3) were proven to be prognostic factors for HNC (Table 2). With the five prognostic DMG models, the risk score value was estimated by the following formula: (0.386 ×   expression level of EPHX3) + (1.388 × expression level of PAX9) + (1.253 × expression level of STK33) + (−0.287 × expression level of GPR150) + (0.342 × expression level of INSM1). According to the median cut‐off point of PI, we divided patients into two groups (high vs. low‐risk group) (Figure 3). Kaplan–Meier curves are presented in Figure 4. The finding suggested that patients with a high‐risk index exhibited worse OS than patients with a low‐risk index (median OS 1.45 months vs. 1.65 years). The results derived from the univariate Cox hazard regression analysis indicated that the HR of the risk score model was 2.53 (95% CI, 1.56–4.09). Furthermore, the multivariate Cox regression analysis also showed consistent findings adjusted for other clinical factors (HR, 2.38; 95% CI, 1.34–4.22).

**Table 2:** Univariate and multivariate Cox analysis for five DMGs

DMGs	Univariate Cox analysis				Multivariate Cox analysis			
	HR	95% CI		Pr( > |*z*|)	HR	95% CI		Pr( > |*z*|)
		lower	Upper			Lower	Upper	
EPHX3	4.359291	1.328137	13.00132	0.0162	1.471	0.3250	6.657	0.616
STK33	7.314855	1.839239	18.74789	0.002738	3.501	0.7821	15.673	0.101
GPR150	3.383151	1.192838	8.170795	0.028524	0.750	0.1804	3.120	0.693
PAX9	7.302176	1.678339	21.22599	0.005578	4.008	0.8622	18.634	0.077
INSM1	7.27867	1.06864	27.27356	0.03997	1.407	0.1873	10.569	0.740

Abbreviations: CI, confidence interval; DMGs, differential methylation genes; HR, hazard ratio.

**Figure 5:**
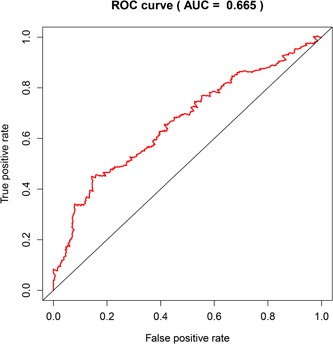
Time‐dependent ROC curve analysis of 5‐year survival prediction by the five key DMGs. AUC, area under the curve; DEGs, differentially expressed genes; ROC, receiver operating characteristic [Color figure can be viewed at wileyonlinelibrary.com]

The OS of the high‐risk group was shorter than that of the low‐risk group. At 3‐ and 5‐year points, the survival rate was 0.53 (0.46–0.61) and 0.40 (0.31–0.52) in the high‐risk group, while in the low‐risk group, the survival rates at 3‐ and 5‐year points was 0.66 (0.59–0.74) and 0.55 (0.46–0.65). Time‐dependent ROC curves were used to assess the prognostic strength based on five‐DMG biomarkers. In the 5‐year OS, the area under the curve of the 5‐DMG model was 0.665 (Figure 5).

Next, the univariate and multivariate Cox regression analysis between clinical information and HNC prognosis was used to verify the prognostic significance. The results indicated that the clinical features of age, gender, pathologic N stage, pathologic T stage, pathologic TNM stage, human papillomavirus (HPV) status by P 16 testing and the five DMG risk score model obviously correlated with OS in the univariate Cox regression analysis. However, clinical covariates of the five DMG risk score model, Age and Radiation were correlated with OS in the multivariate Cox regression analysis (Table 3).

**Table 3:** Univariate and multivariate Cox analysis for clinical variables

Variables	Univariate Cox analysis				Multivariate Cox analysis			
	HR	95% CI		Pr( > |*z*|)	HR	95% CI		Pr( > |*z*|)
		Lower	Upper			Lower	Upper	
Age	1.020483	1.007544	1.033587	0.001843	1.0172	1.0034	1.0311	0.01412
Gender	1.440396	1.073269	1.933104	0.015057	1.1838	0.8627	1.6243	0.29597
Grade	0.9675	0.825366	1.134111	0.683598	1.0677	0.8786	1.2976	0.50995
History of alcohol consumption	0.873621	0.681775	1.119453	0.285523	1.0468	0.8036	1.3635	0.73469
History of neoadjuvant treatment	1.626489	0.825141	3.206076	0.160062	0.7971	0.3375	1.8828	0.6051
History of tobacco smoking	1.282177	0.930753	1.766289	0.128291	1.2483	0.9048	1.7222	0.17674
HPV status by P 16 testing	1.232958	1.039454	1.462484	0.016207	1.0895	0.9136	1.2992	0.34012
Pathological TNM stage	1.212737	1.057586	1.39065	0.005752	1.2012	0.9897	1.458	0.06353
M stage	1.133245	0.970127	1.323789	0.114684				
N stage	1.175107	1.073757	1.286024	0.000454				2
T stage	1.153662	1.026341	1.296778	0.016587				
New tumor events event after initial treatment	1.177114	0.987978	1.402456	0.068057	1.2872	0.9144	1.8119	0.14788
Person neoplasm cancer status	2.629388	2.218622	3.116205	6.79E‐29	2.5028	2.0703	3.0256	2.00E‐16
Race	1.091668	0.960811	1.240346	0.178203	1.0657	0.9298	1.2214	0.36065
Radiation	0.999551	0.808251	1.236128	0.996693	0.6584	0.4634	0.9353	0.01961
Risk score of five genes	2.525086	1.556294	4.096949	0.000176	2.3822	1.345	4.2193	0.00292

Abbreviations: CI, confidence interval; HR, hazard ratio.

### 3.6 Functional enriched analysis based on the five DMG model

**Figure 6:**
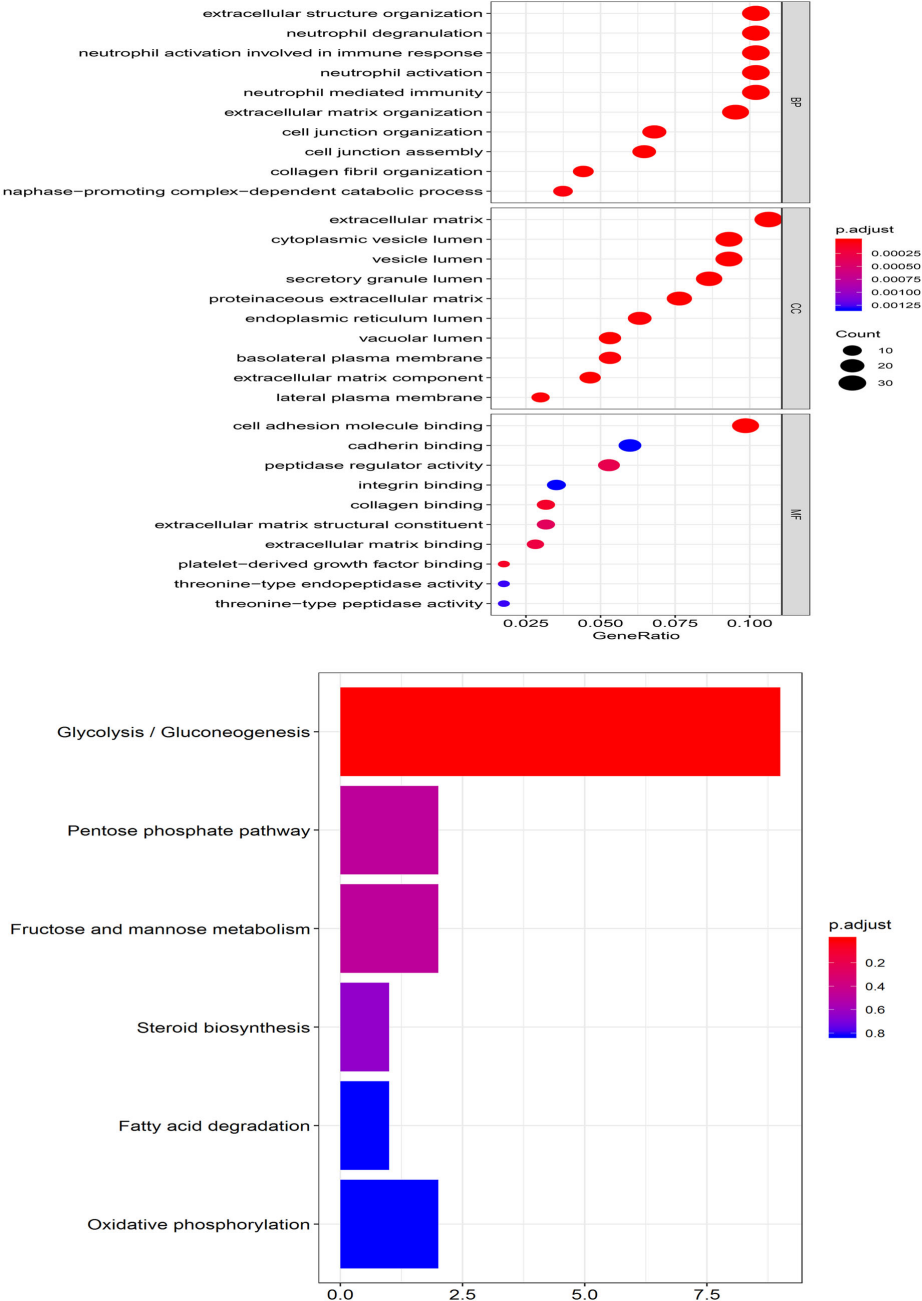
Functional enrichment analysis based on the five differential methylation gene models [Color figure can be viewed at wileyonlinelibrary.com]

We performed differential expressed analysis (FDR < 0.05) between low and high‐risk groups based on the five DMG models in the TCGA HNC cohort, and the top 200 genes (100 upregulated genes and 100 downregulated genes) were selected. The enrichment analysis was used to explore the biological functions of the DEGs. The results demonstrated that 509 Gene Ontology (GO) terms were observed in the biological process, such as collagen fibril organization (GO:0030199), positive regulation of the canonical Wnt signaling pathway (GO:0090263), extracellular matrix (GO:0031012), and cell adhesion molecule binding (GO:0050839). Six KEGG pathways were detected, including the Pentose phosphate pathway (hsa00030], steroid biosynthesis [hsa00100], fatty acid degradation (hsa00071), and oxidative phosphorylation (hsa00190). The GO and KEGG functional analysis is shown in Figure 6.

**Figure 7:**
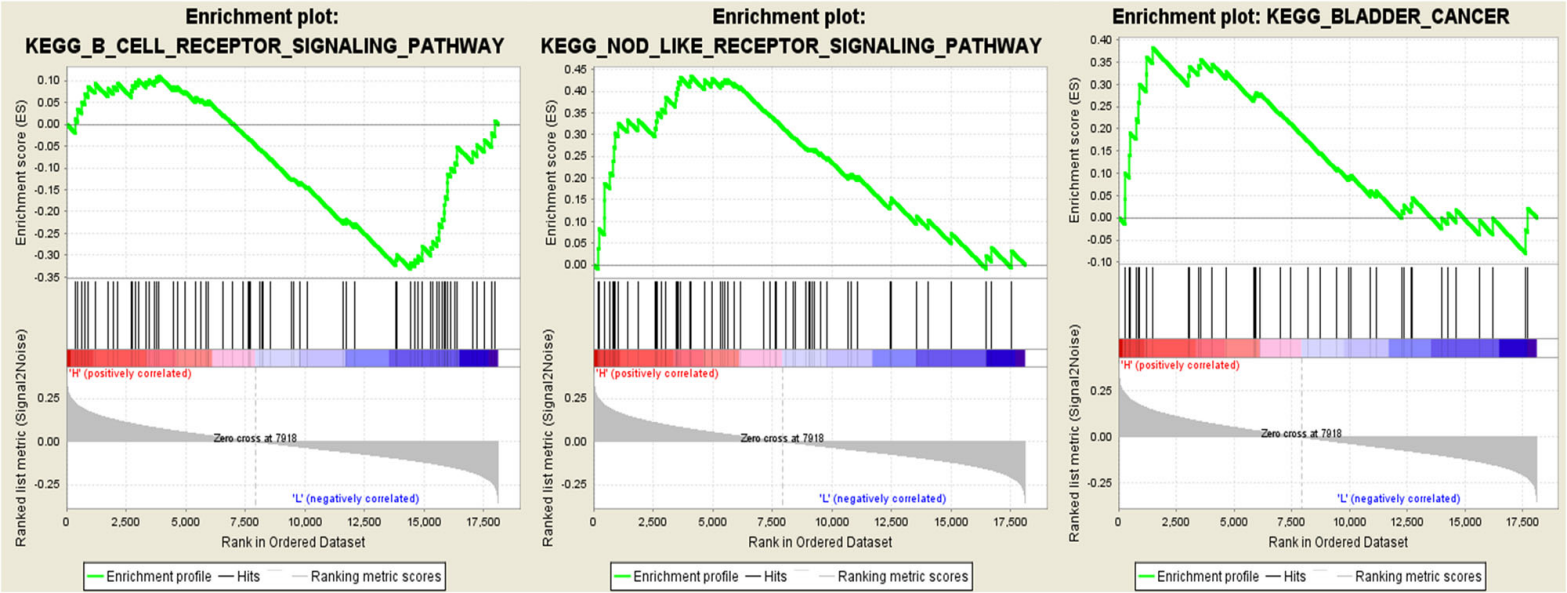
The top four enriched pathways in the high‐risk group analyzed by gene set enrichment analysis [Color figure can be viewed at wileyonlinelibrary.com]

GSEA demonstrated that 86 of 177 gene sets were upregulated in the highly expressed phenotype group, and 91/177 gene sets were upregulated in the low phenotype expression group. The high‐risk groups have increased expression of NOD‐like receptor signaling pathways and RIG I like receptor signaling pathways (Figure 7).

### 3.7 Prognostic OS assessment of DNA methylation sites in five DMG models

**Figure 8:**
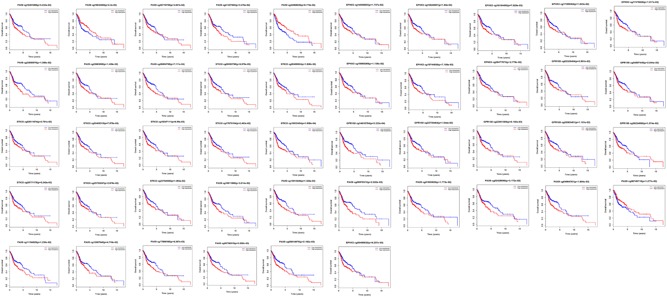
Kaplan–Meier survival curves for overall survival according to the risk cutoff point for prognostic DNA methylation sites [Color figure can be viewed at wileyonlinelibrary.com]

Data relating to DNA methylation sites in five DMGs were obtained from the TCGA HNC cohort. Then, the univariate Cox regression analysis was performed to select the prognosis DNA methylation site, and the results demonstrated that 51 DNA methylation sites in five DMGs obviously correlated with OS in patients with HNC (Table 2). Kaplan–Meier survival analysis was further used to examine the results. The findings indicated that 46 prognostic DNA methylation sites were observed (Figure 8). The overlapping 41 hub DNA methylation sites were identified.

### 3.8 Correlation analysis between abnormal mRNAs and DNA methylation sites in HNC

**Figure 9:**
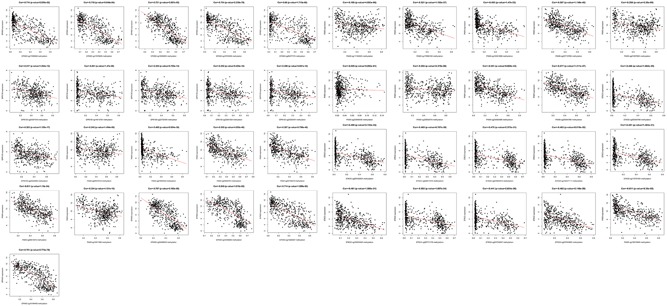
Correlation analysis between gene expression and DNA methylation sites. The abscissa is the beta value of DNA methylation sites, and the ordinate is the gene expression level [Color figure can be viewed at wileyonlinelibrary.com]

The key DNA methylation sites were used to explore the correlation between mRNAs and DNA methylation sites. The negative correlations between DNA methylation sites and mRNAs were a matter of concern. The 41 DNA methylation sites were identified to negatively correlate with mRNAs, and the r‐value in the Pearson's correlation analysis in 34 DNA methylation sites was < − 0.3 (Figure 9).

### 3.9 Prognostic analysis between hypermethylation/hypomethylation and mRNA expression in HNC

**Figure 10:**
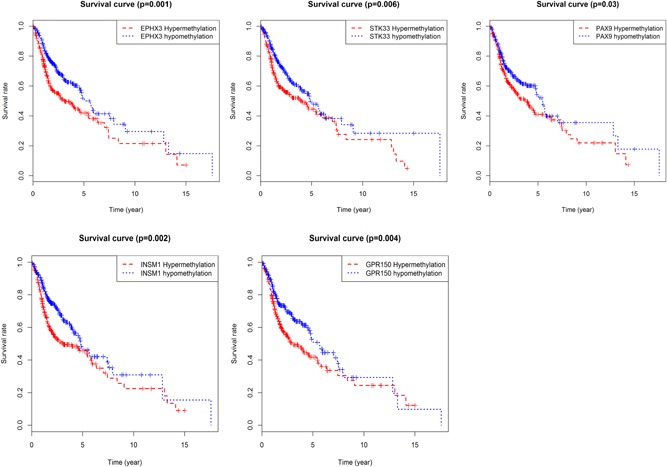
Kaplan–Meier survival analysis between hypermethylation and hypomethylation in five differentially expressed genes [Color figure can be viewed at wileyonlinelibrary.com]

**Figure 11:**
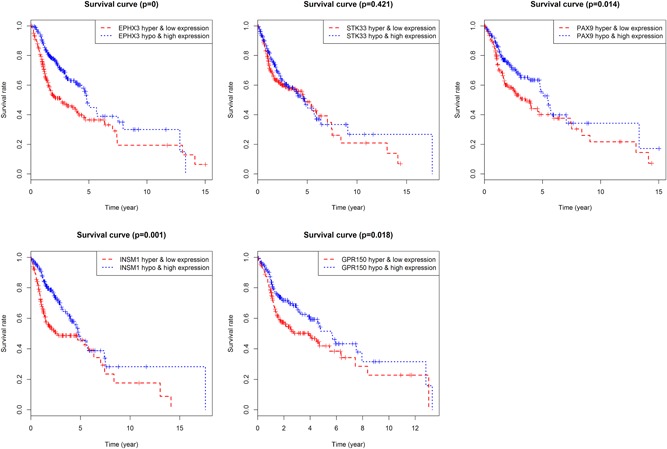
Survival comparison between gene hypermethylation and low expression as well as gene hypomethylation and high expression in five differentially expressed genes [Color figure can be viewed at wileyonlinelibrary.com]

The five DMGs with hypermethylation or hypomethylation was conducted along with Kaplan–Meier survival analysis, and the results indicated that PAX9, STK33, GPR150, INSM1, and EPHX3 with hypermethylation or hypomethylation were positively associated with OS (Figure 10). Patients with hypomethylation of PAX9, STK33, GPR150, INSM1, and EPHX3 showed a shorter survival time and a higher tumor‐related mortality. PAX9, GPR150, INSM1, and EPHX3 with hypermethylation and low expression and hypomethylation and high expression were also significantly associated with OS, with the exception of EPHX3 (Figure 11).

### 3.10 Establishment and validation of a prognostic nomogram model

**Figure 12:**
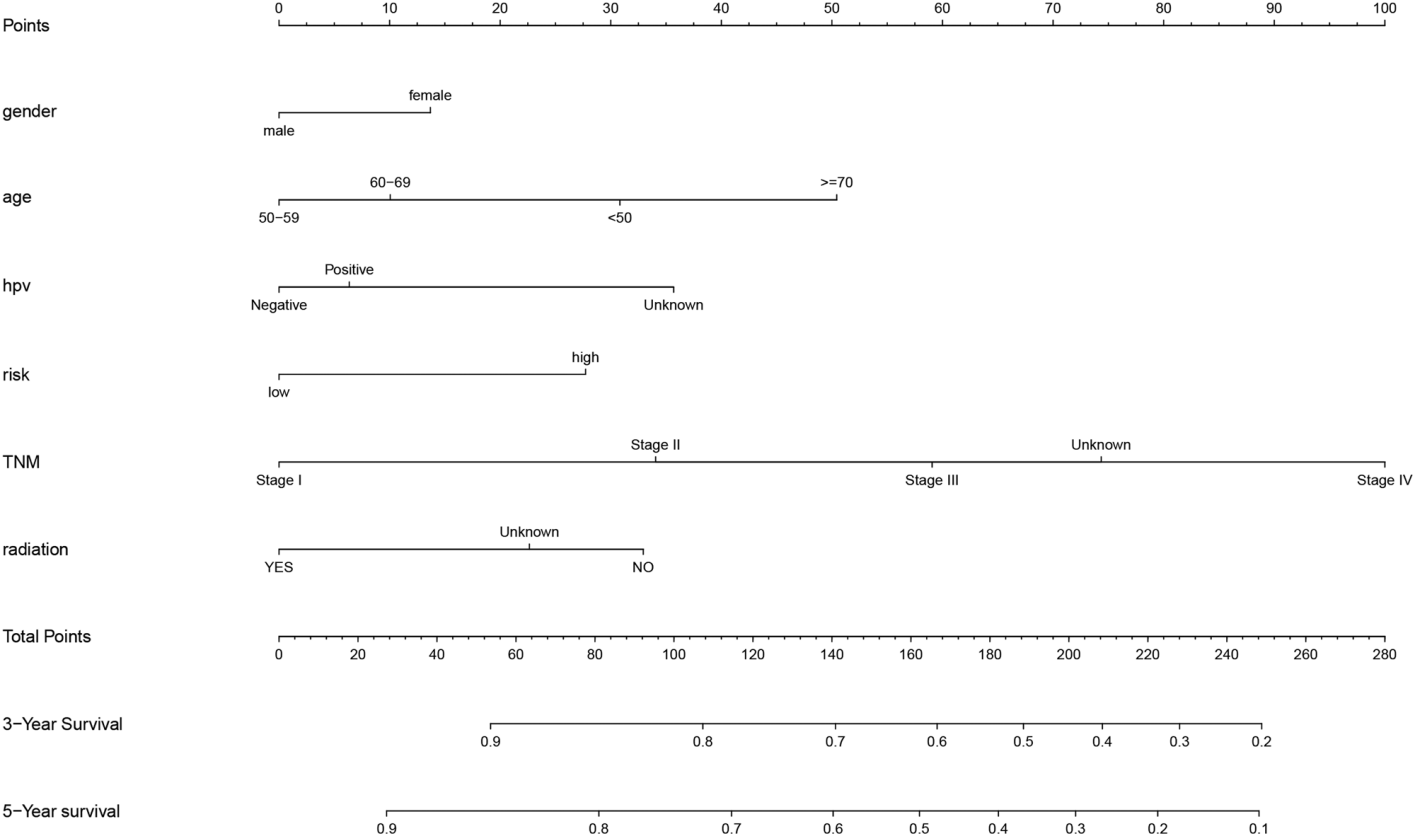
Nomograms for estimating 3‐ and 5‐year overall survival survival

Obvious variables screened by the Cox regression analysis were used to establish a nomogram model to forecast the probability of OS in the TCGA Head–Neck Squamous Cell Carcinoma (HNSC) cohort (Figure 12). The gender, age, HPV status by P 16 testing, a risk score model of five genes, pathological TNM Stage, and radiation were considered in the nomogram. The nomogram suggested that pathological TNM Stage and age and HPV status by P 16 testing were the biggest contributors to prognosis, while gender was a lower contributor.

**Figure 13:**
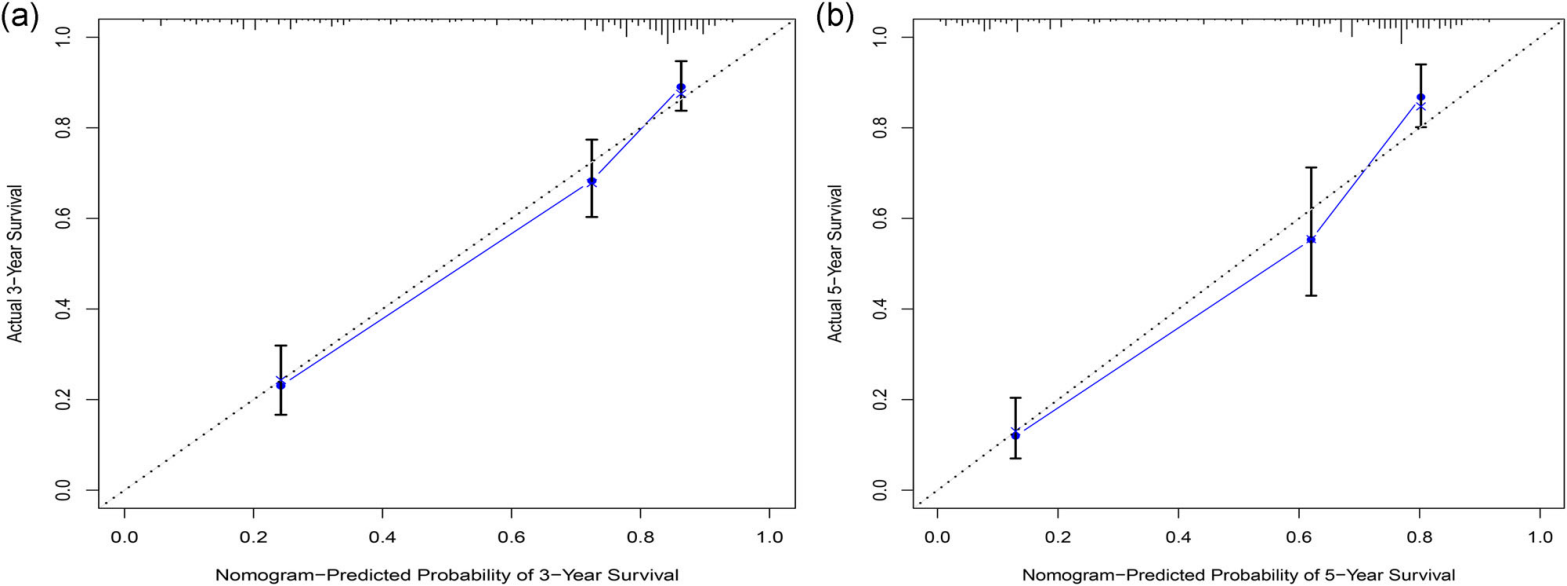
3‐ (a) and (b) 5‐year overall survival calibration curves

We used the bootstrap method to validate the results of the nomogram model. The results showed the nomogram could correctly predict for OS with a C‐index of 0.767 (95% CI, 0.730–0.803). The calibration chart showed good consistency between the nomogram prediction and the actual observation of the 3‐ and 5‐year OS rates (Figure 13).

### 3.11 Validation of hub genes with GEO data set

**Figure 14:**
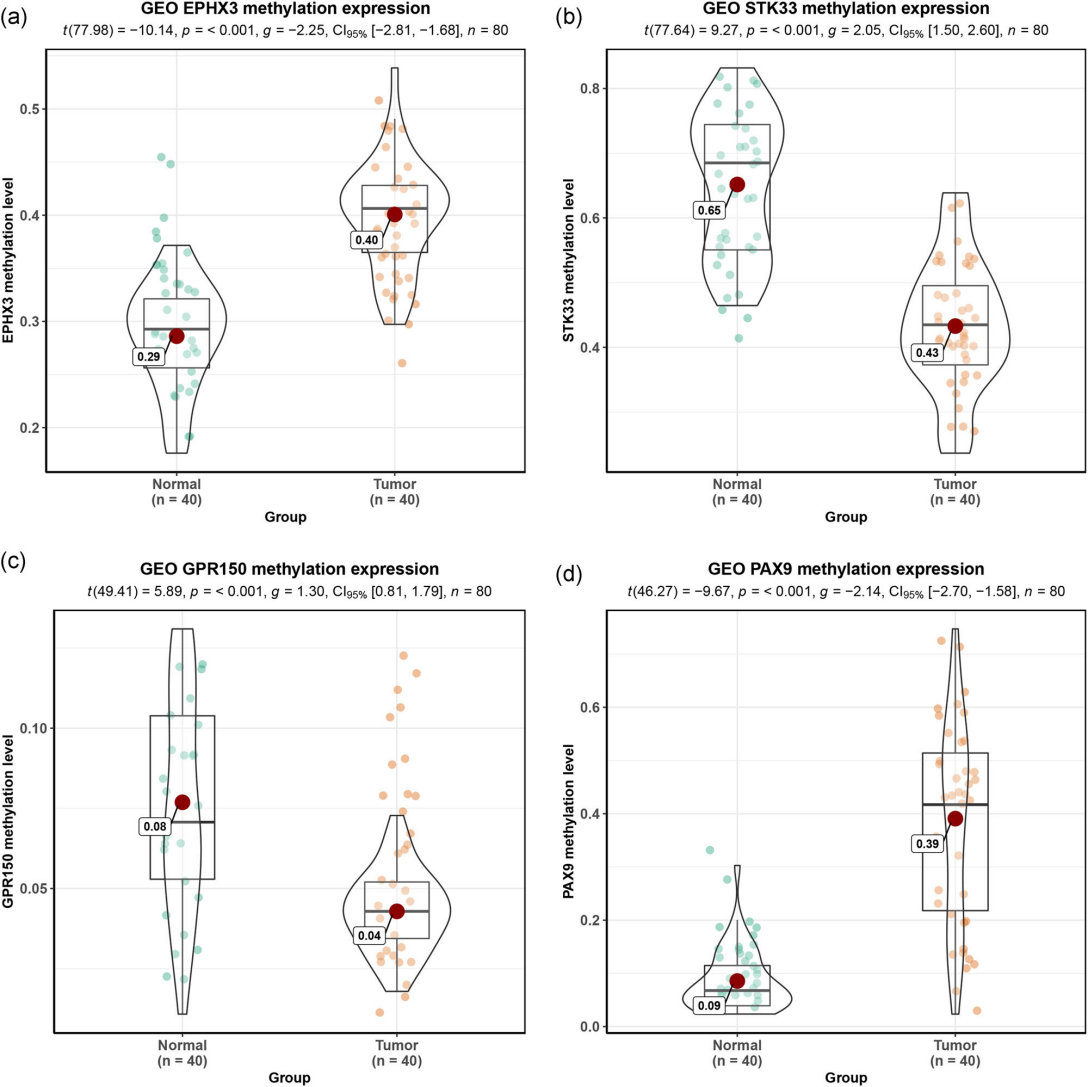
The methylation level of four hub genes in GSE38532. (a) EPHX3; (b) STK33; (c) GPR150 and (d) PAX9 [Color figure can be viewed at wileyonlinelibrary.com]

One study (GSE38532) was included in the GEO data set. Analysis of mRNA expression profiles identified a total of 11481 DMGs. Among these genes, 5,686 genes were defined as hypermethylation genes, and 5,795 genes were regarded as hypomethylation genes. Four hub genes (EPHX3, STK33, GPR150, and PAX9) were detected in the GSE38532 data set. The four DMG methylation level in the GSE38532 is observed in Figure 14.

## 4 DISCUSSION

HNC is noted for its rapid clinical progress and poor prognosis. In the past 40 years, its survival rate has hardly improved. The primary common pathological subtype is squamous cell carcinoma (SCC), which makes up about 90% of all HNC cancers (Boonkitticharoen et al., 2008). Multiple factors might contribute to HNC including smoking and drinking, obesity, insufficient physical exercise, viral infection, especially infection of the Epstein–Barr virus and HPV (Hennessey, Westra, & Califano, 2009; Lo et al., 2006). Early HNC patients have blurred symptoms and minimal body changes; early symptoms of cancer are often difficult to find. Despite efforts to screen molecular biomarkers for early detection and efforts to develop new treatment methods, HNC prognosis remains not ideal. Local recurrence and metastasis are primary risk factors in our efforts to achieve a satisfactory treatment effect of HNC (Le, Squarize, & Castilho, 2014; Molinolo et al., 2009; Papillon‐Cavanagh et al., 2017). Therefore, as a solution, identifying molecular biomarkers of HNC remains urgent.

DNA methylation is a heritable and stable epigenetic modification that usually occurs at the CpG site, and it plays an important role in regulating protein function, gene expression, and RNA processing. Abnormal promoter methylation is thought to be the primary mechanism for tumor‐associated gene inactivation (Baylin, 2005; Portela & Esteller, 2010). DNA methylation has also been investigated in cancer and likely results in genome rearrangement and chromosomal instability. Several studies indicate that abnormal accumulation of genetic and epigenetic modification plays a crucial role in tumor occurrence (Allen, Pezone, Porcellini, Muller, & Masternak, 2017; Duruisseaux et al., 2018; Nagata et al., 2012). A previous study by Zhou et al. (2018) demonstrated that DNA methylation may serve as a tool to screen and identify a valuable biomarker for the prediction of prognosis in patients with HNC. Hypermethylation of the SEMA3B promoter region was observed in oral SCC (OSCC) tissues, and an obvious difference in the methylation frequency of the gene was detected between OSCC and noncancerous samples (Wang, Ling, Wu, & Zhang, 2013). A study by Hwang et al. (2013) reported that the promoter methylation status of GLT8D1 and C6orf136 induced by aberrant upregulation of FOXM1 may be exploitable for clinical use as early biomarkers of cancer predisposition. Another study showed that HPV infection significantly influences DNA methylation at different anatomical regions in the TCGA HNSC cohort (Degli Esposti et al., 2017). Therefore, the promoter DNA methylation played a vital role in the development of carcinogenesis. However, it is rarely reported why there is a correlation between the methylation of genes and the development of HNC. Therefore, we aimed to explore their potential roles as molecular biomarkers in the evaluation of HNC development as part of our work. Data regarding gene expression and gene methylation profiles were obtained from the TCGA data set. By analyzing the DNA methylation profiles of patients combined with the analysis result of gene expression data, we found that 324 DMGs and 7,299 DEGs were found during differential analysis. A total of 130 DMGs/675 DEGs significantly correlated with OS when univariate COX regression analysis was performed. The common 21 genes were screened by overlapping 129 DMGs and 674 DEGs. Five negative association pairs between methylation genes and mRNA (PAX9: ENSG00000198807, STK33: ENSG00000130413, GPR150: ENSG00000178015, INSM1: ENSG00000173404, and EPHX3: ENSG00000105131) were identified. By Cox regression analysis, five DMGs and several methylation sites were identified as potential prognostic molecular biomarkers. Regarding the five DMGs, a risk score model was established. Patients were stratified into low‐risk and high‐risk groups based on the five DMG prognostic model. Patients in the low‐risk group had a higher OS than those in the high‐risk group. The functional enrichment analysis showed that the mRNA profiles based on the five DMG models were mostly enriched in pathways pertaining to the extracellular matrix, cell adhesion, and immune responses. The GSEA analysis also revealed that pathways including the RIG I like receptor signaling pathway and the NOD‐like receptor signaling pathway were involved in the development of HNC. Several DNA methylation sites in five DMGs models were also found and associated with the prognosis of HNC, such as PAX9 cg04994761, STK33 cg18933494, EPHX3 cg19744936, GPR150 cg25583491, and GPR150 cg22730464. By performing Kaplan–Meier survival analysis, we found that the five DMGs with hypermethylation or hypomethylation were associated with poorer OS in HNC.

Epigenetic modifications, such as DNA methylation in gene promoters, often inhibit gene transcription and protein translation, and it exerts an important risk factor in human carcinogenesis. Several studies have demonstrated that DNA methylation is regarded as an early event of tumor development, and new work focuses on screening biomarkers for early tumor detection, selection of treatment options, and accurate prognosis, especially in HNC (Sasidharan Nair, Toor, Taha, Shaath, & Elkord, 2018; Yim et al., 2018; Zhou et al., 2018). The underlying mechanisms that elucidate the development and progression of HNC will certainly greatly benefit diagnostic, therapeutic, and prognostic assessments. Rani et al analyzed the integration of DNA methylation and mRNA data and indicated that the alteration in DNA methylation is associated with the mRNA expression of the PAX9 gene, which allows for risk stratification of early stage chronic lymphocytic leukemia patients (Rani et al., 2017). Yin, Ma, Liu, and Chen (2018) indicated that STK33 hypermethylation is considered to be a promising new biomarker for the diagnosis, prognosis, and treatment of CRC. Cai et al conducted genome‐wide screening and found that abnormal methylation of GPR150, ITGA8, and HOXD11 could be used as a tumor marker (Cai et al., 2007). The aberrant expression of INSM1 could be found in various cancers, such as head and neck tumors (Rooper, Bishop, & Westra, 2018), neuroendocrine carcinoma (Kuji et al., 2017), Merkel cell carcinoma (Lilo, Chen, & LeBlanc, 2018), and small cell lung cancer (McColl et al., 2017). A study by Morandi et al. (2017) showed that hypermethylation of EPHX3 might contribute to the development of OSCC. A study by Bell (2011) used microarray analysis in patients with salivary gland adenoid cystic carcinoma for aberrant DNA methylation, and the results suggested that aberrant DNA methylation of EPHX3 is associated with adenoid cystic carcinoma development and progression.

## 5 CONCLUSION

In our study, five DMGs (PAX9, STK33, GPR150, INSM1, and EPHX3) and several methylation sites were our primary concern for HNC. These findings help us to speculate on the prognosis of patients with HNC. In addition, these results provide new perspectives in exploring the molecular mechanisms of DMG and site‐specific methylation of HNC development.

## CONFLICT OF INTERESTS

The authors declare that there are no conflict of interests.

## AUTHOR CONTRIBUTIONS

J.K.S., G.H.B., Y.W.Y., and C.Z. wrote the main manuscript text; J.K.S., Y.T., and X.H.Y. prepared Figures 1–14; J.K.S. and G.H.B. contributed to data analysis. All authors reviewed the manuscript.

## DATA AVAILABILITY

Data availability could be obtained from the TCGA website.

## Supporting information

Supporting information

Supporting information
