# Supplementary material for: Systematic analysis of differentially methylated expressed genes and site‐speciﬁc methylation as potential prognostic markers in head and neck cancer
Source: J Cell Physiol. 2019 May 26;234(12):22687–702. doi: 10.1002/jcp.28835 (PMC6772109; doi:10.1002/jcp.28835)
Supplement: Supplementary file 1 — Supporting information [file JCP-234-22687-s001.docx]

Supplementary Table 1 Univariate Cox analysis for DMGs.

| gene | HR | z | P-value |
| --- | --- | --- | --- |
| P | 0.158844 | -2.08267 | 0.037282 |
| AC091199.1 | 2.41439 | 2.182488 | 0.029074 |
| AC006942.4 | 3.464601 | 2.034112 | 0.04194 |
| AC026904.1 | 0.216607 | -2.532 | 0.011341 |
| RP11-98O2.1 | 2.857347 | 2.444627 | 0.0145 |
| GPS2P2 | 2.500722 | 2.326498 | 0.019992 |
| AC003682.17 | 0.022165 | -2.65111 | 0.008023 |
| PIK3IP1-AS1 | 161.3168 | 2.18506 | 0.028884 |
| SLITRK1 | 0.331416 | -2.17553 | 0.02959 |
| CCT6P3 | 0.000144 | -3.92218 | 8.78E-05 |
| SLC2A10 | 5.624204 | 2.478193 | 0.013205 |
| CTSW | 1357.862 | 3.445604 | 0.00057 |
| MIR106A | 2.235286 | 2.599845 | 0.009327 |
| RP3-414A15.10 | 11.71854 | 3.559432 | 0.000372 |
| ALKBH3-AS1 | 0.227576 | -2.5624 | 0.010395 |
| SLC22A16 | 4.07678 | 2.073972 | 0.038082 |
| FKSG48 | 2.206852 | 2.70376 | 0.006856 |
| RP11-396B14.2 | 0.058129 | -2.19128 | 0.028431 |
| NKX2-3 | 3.299265 | 1.989916 | 0.0466 |
| RARRES2P1 | 0.16908 | -2.30533 | 0.021148 |
| RP11-114B7.6 | 0.140115 | -2.58744 | 0.009669 |
| HOXA-AS2 | 0.202439 | -2.02765 | 0.042596 |
| LINC01471 | 41.76754 | 3.213666 | 0.001311 |
| SIM2 | 4.707555 | 2.208486 | 0.02721 |
| RPS20P35 | 0.347945 | -2.96396 | 0.003037 |
| KLK2 | 12.98319 | 2.835662 | 0.004573 |
| RP11-1006G14.2 | 3.606365 | 2.94099 | 0.003272 |
| GDF7 | 6.179932 | 2.338102 | 0.019382 |
| SIRPG | 8.396417 | 2.569674 | 0.010179 |
| ZNF479 | 0.018584 | -2.70245 | 0.006883 |
| HIST1H3E | 0.16502 | -2.59457 | 0.009471 |
| TMCO5B | 0.184977 | -2.61786 | 0.008848 |
| STON1-GTF2A1L | 0.005155 | -3.93298 | 8.39E-05 |
| HOXB13-AS1_2 | 2.26564 | 2.184341 | 0.028937 |
| AC021660.1 | 9.590518 | 2.539033 | 0.011116 |
| RP11-130C6.1 | 5.835503 | 2.031543 | 0.0422 |
| HIST1H4A | 3.725499 | 2.527277 | 0.011495 |
| SCGB3A1 | 8.170773 | 3.039228 | 0.002372 |
| LINC00883 | 72.84147 | 2.150022 | 0.031553 |
| RP4-529N6.1 | 17.88321 | 2.884585 | 0.003919 |
| MIR514A3 | 0.367418 | -2.05277 | 0.040095 |
| RP11-540O11.1 | 0.042184 | -2.25886 | 0.023892 |
| FBXL8 | 16.85976 | 2.719363 | 0.006541 |
| EPHX3 | 4.155424 | 2.447581 | 0.014382 |
| TNFSF13B | 5.568027 | 2.144863 | 0.031964 |
| CTD-2521M24.5 | 11.75849 | 2.487895 | 0.01285 |
| AC130469.1 | 8.462469 | 3.130202 | 0.001747 |
| RP11-886D15.1 | 0.117409 | -2.69004 | 0.007144 |
| RP11-395F4.1 | 2.919318 | 2.28563 | 0.022276 |
| ZSCAN31 | 2.196236 | 2.028471 | 0.042512 |
| LINC01574 | 8.711992 | 2.683904 | 0.007277 |
| RP11-3B12.2 | 7.106091 | 1.965243 | 0.049386 |
| RP11-209K10.2 | 5.728695 | 2.404362 | 0.016201 |
| STK33 | 5.872125 | 2.988773 | 0.002801 |
| TBX5-AS1 | 2.466236 | 2.005125 | 0.04495 |
| RP11-15B24.5 | 3839.766 | 2.170703 | 0.029954 |
| RN7SL653P | 0.227147 | -2.85127 | 0.004354 |
| SLAMF6 | 5.338654 | 2.206695 | 0.027335 |
| AC016735.1 | 180.8045 | 2.607295 | 0.009126 |
| RP11-231N3.1 | 2.973061 | 2.759937 | 0.005781 |
| ZSCAN23 | 0.3257 | -1.96409 | 0.04952 |
| GPR150 | 3.121928 | 2.319183 | 0.020385 |
| CTC-444N24.13 | 0.067629 | -1.97351 | 0.048438 |
| AK4P5 | 2.069252 | 2.029605 | 0.042397 |
| RP5-1120P11.3 | 1.96826 | 2.121234 | 0.033902 |
| CTC-471J1.11 | 5.446428 | 2.283791 | 0.022384 |
| MAP3K14-AS1 | 2.327872 | 1.996851 | 0.045841 |
| DUXAP3 | 4.406907 | 2.446392 | 0.014429 |
| CSTA | 0.207592 | -2.4713 | 0.013462 |
| AC079790.2 | 11.8896 | 2.401041 | 0.016349 |
| AP000697.6 | 8.621358 | 2.916068 | 0.003545 |
| TLX2 | 5.139015 | 1.967355 | 0.049142 |
| AC005514.2 | 0.241573 | -2.23888 | 0.025163 |
| AC078883.3 | 0.375569 | -2.83618 | 0.004566 |
| CTD-3195I5.3 | 0.429435 | -2.25258 | 0.024286 |
| CMTM3 | 4.701165 | 2.251689 | 0.024342 |
| SP9 | 2.868296 | 2.846257 | 0.004424 |
| AC019011.1 | 4.960723 | 3.031949 | 0.00243 |
| RP11-848P1.7 | 3.405791 | 2.548745 | 0.010811 |
| MIR891A | 0.173502 | -2.03522 | 0.041828 |
| CLGN | 3.292339 | 2.344972 | 0.019029 |
| VN1R2 | 0.085093 | -2.05353 | 0.040021 |
| RP11-4B14.3 | 2.614263 | 2.684117 | 0.007272 |
| PNMAL1 | 4.621739 | 2.614677 | 0.008931 |
| VPREB1 | 8.396919 | 2.251842 | 0.024332 |
| MIAT | 8.895009 | 2.08835 | 0.036766 |
| CTD-2540M10.1 | 3.205684 | 3.392382 | 0.000693 |
| WNK3 | 4.407958 | 3.225314 | 0.001258 |
| SMIM7 | 6.568683 | 3.543313 | 0.000395 |
| GS1-72M22.1 | 0.272761 | -2.83845 | 0.004533 |
| NEUROD2 | 9.356562 | 2.245508 | 0.024736 |
| CEMP1 | 33.95887 | 2.897969 | 0.003756 |
| AC005624.2 | 0.00596 | -3.23575 | 0.001213 |
| KRTAP3-3 | 50.50617 | 2.772292 | 0.005566 |
| EIF3K | 40.318 | 2.426607 | 0.015241 |
| PAX9 | 5.968618 | 2.759893 | 0.005782 |
| SRY | 0.479221 | -2.62861 | 0.008573 |
| RNA5SP38 | 0.261452 | -3.06549 | 0.002173 |
| SNORD114-29 | 0.314289 | -2.69695 | 0.006998 |
| SPANXN4 | 0.226828 | -2.09136 | 0.036496 |
| GRP | 0.224806 | -2.04581 | 0.040775 |
| AC007906.1 | 0.383995 | -2.7931 | 0.005221 |
| PARVG | 5.517004 | 2.022289 | 0.043146 |
| MIR150 | 14.83603 | 3.071917 | 0.002127 |
| RNU6-502P | 18.35876 | 2.479559 | 0.013154 |
| LINC00424 | 0.107672 | -1.99319 | 0.04624 |
| EBI3 | 991.1615 | 3.35345 | 0.000798 |
| RP11-15K2.2 | 3.33691 | 2.745515 | 0.006042 |
| KCNC2 | 0.141524 | -2.51177 | 0.012013 |
| RP11-626H12.1 | 5.57742 | 2.115108 | 0.034421 |
| MMP23B | 2.457251 | 2.499852 | 0.012425 |
| LINC00944 | 12.97844 | 2.755551 | 0.005859 |
| RP11-445F12.1 | 5.025047 | 2.526152 | 0.011532 |
| INSM1 | 5.398667 | 2.040294 | 0.041321 |
| C6orf183 | 9.188855 | 2.937701 | 0.003307 |
| LRRC34 | 8.097723 | 3.077629 | 0.002087 |
| RP11-24M17.4 | 129.9861 | 3.256677 | 0.001127 |
| RP11-982M15.7 | 37.82493 | 2.497165 | 0.012519 |
| CTD-3065J16.6 | 2.643684 | 2.084558 | 0.037109 |
| KB-1410C5.5 | 2.574478 | 2.103335 | 0.035437 |
| RP11-278H7.4 | 4.876142 | 2.091095 | 0.03652 |
| U1 | 0.139666 | -2.61093 | 0.00903 |
| RNU2-37P | 2.123721 | 2.056729 | 0.039712 |
| CTB-118N6.2 | 0.072151 | -2.6024 | 0.009257 |
| RP11-259G18.1 | 25.40188 | 3.354631 | 0.000795 |
| RNA5SP382 | 8.297304 | 2.400963 | 0.016352 |
| CTD-2161F6.3 | 0.328712 | -2.09989 | 0.035739 |
| MIR3622B | 3.084222 | 2.044041 | 0.04095 |
| DGAT2L7P | 3.912629 | 2.297226 | 0.021606 |
| TCN1 | 4.105709 | 2.425311 | 0.015295 |
