# Supplementary material for: Systematic analysis of differentially methylated expressed genes and site‐speciﬁc methylation as potential prognostic markers in head and neck cancer
Source: J Cell Physiol. 2019 May 26;234(12):22687–702. doi: 10.1002/jcp.28835 (PMC6772109; doi:10.1002/jcp.28835)
Supplement: Supplementary file 2 — Supporting information [file JCP-234-22687-s002.docx]

Supplementary Table 2 Univariate Cox analysis for DEGs.

| gene | HR | z | P-value |
| --- | --- | --- | --- |
| PLIN1 | 1.064097 | 2.1622 | 0.030603 |
| EMP1 | 1.119071 | 2.436888 | 0.014814 |
| NUCB2 | 1.192649 | 2.633927 | 0.00844 |
| CIDEC | 1.069828 | 2.193802 | 0.02825 |
| CAB39L | 1.156688 | 2.203632 | 0.02755 |
| HTN3 | 1.04744 | 2.488277 | 0.012836 |
| RSPH1 | 1.111131 | 2.626089 | 0.008637 |
| PIFO | 1.13513 | 3.203887 | 0.001356 |
| C1orf189 | 1.131832 | 2.174817 | 0.029644 |
| KRTAP13-1 | 1.098128 | 1.997427 | 0.045779 |
| TMEM232 | 1.119372 | 2.043894 | 0.040964 |
| KRTAP3-3 | 1.123587 | 2.32643 | 0.019996 |
| CFTR | 1.050257 | 2.095303 | 0.036144 |
| KRTAP4-12 | 1.219949 | 2.060769 | 0.039325 |
| ADH1B | 1.045649 | 2.080602 | 0.03747 |
| ATP6V0A4 | 1.06187 | 2.23213 | 0.025606 |
| CAPN5 | 1.130544 | 2.390096 | 0.016844 |
| KRTAP1-3 | 1.193749 | 2.305333 | 0.021148 |
| CFAP52 | 1.157763 | 2.79993 | 0.005111 |
| CYP3A4 | 1.095642 | 2.431483 | 0.015037 |
| ACTN3 | 0.927591 | -1.97351 | 0.048437 |
| KRT4 | 1.037056 | 2.386428 | 0.017013 |
| PHYH | 1.140653 | 2.312112 | 0.020772 |
| KRTAP4-2 | 1.280831 | 2.846168 | 0.004425 |
| KRTAP2-2 | 1.320426 | 3.602884 | 0.000315 |
| PFKM | 1.14457 | 2.179323 | 0.029308 |
| TIMP4 | 1.142615 | 3.708442 | 0.000209 |
| ADPRHL1 | 1.122033 | 2.808738 | 0.004974 |
| KRTAP4-4 | 1.247418 | 2.150752 | 0.031496 |
| KRTAP10-7 | 1.253141 | 2.372841 | 0.017652 |
| GMDS | 1.224457 | 2.729763 | 0.006338 |
| ANKRD66 | 1.110091 | 2.021083 | 0.043271 |
| CAPSL | 1.113908 | 2.862012 | 0.00421 |
| PKLR | 0.923166 | -1.97337 | 0.048454 |
| KRTAP4-3 | 1.276012 | 2.064048 | 0.039013 |
| UPK3A | 0.907583 | -1.97193 | 0.048618 |
| KRTAP24-1 | 1.339625 | 2.672282 | 0.007534 |
| KRTAP4-5 | 1.295951 | 2.289738 | 0.022037 |
| PPP1R3C | 1.092475 | 2.972845 | 0.002951 |
| C11orf88 | 1.106298 | 2.465254 | 0.013692 |
| FEM1A | 0.802388 | -2.54951 | 0.010787 |
| KRTAP2-1 | 1.287197 | 2.27466 | 0.022926 |
| SNTN | 1.088937 | 2.289706 | 0.022038 |
| GOT1 | 1.199009 | 2.095205 | 0.036153 |
| TRNP1 | 1.155167 | 3.147386 | 0.001647 |
| TYRP1 | 1.05968 | 2.070984 | 0.03836 |
| STOML3 | 1.123981 | 3.13036 | 0.001746 |
| KRTAP10-8 | 1.37046 | 3.097574 | 0.001951 |
| FAM26D | 1.187444 | 2.312643 | 0.020742 |
| SHROOM3 | 1.122459 | 2.229294 | 0.025794 |
| KRTAP3-2 | 1.101317 | 2.396013 | 0.016575 |
| KRTAP4-8 | 1.141932 | 1.99917 | 0.04559 |
| ARMC3 | 1.108205 | 2.746211 | 0.006029 |
| SPATA4 | 1.135571 | 2.474144 | 0.013356 |
| KRTAP7-1 | 1.208291 | 2.285225 | 0.0223 |
| PIH1D3 | 1.124459 | 2.027263 | 0.042635 |
| KRTAP3-1 | 1.11533 | 3.210534 | 0.001325 |
| DRC3 | 0.842657 | -2.03403 | 0.041949 |
| STK39 | 1.226189 | 2.373574 | 0.017617 |
| CSRNP1 | 1.172641 | 2.08188 | 0.037353 |
| COBL | 1.091285 | 2.879154 | 0.003987 |
| ARSF | 1.074749 | 2.550929 | 0.010744 |
| GYS2 | 1.062041 | 2.045588 | 0.040797 |
| TSPAN6 | 1.243615 | 2.8378 | 0.004543 |
| SMAGP | 1.219405 | 2.666272 | 0.00767 |
| MAPKAPK3 | 1.228682 | 2.274765 | 0.02292 |
| ANXA1 | 1.103328 | 2.11085 | 0.034785 |
| WWC1 | 1.181553 | 2.423942 | 0.015353 |
| MYOT | 1.044635 | 2.143406 | 0.032081 |
| CLYBL | 1.158196 | 2.272205 | 0.023074 |
| C20orf85 | 1.080616 | 2.223942 | 0.026152 |
| KLHDC7A | 1.060451 | 2.075073 | 0.03798 |
| AMY2B | 0.90411 | -2.20965 | 0.027129 |
| ANGPTL5 | 1.11642 | 2.747244 | 0.00601 |
| CLDN23 | 1.147132 | 2.359219 | 0.018313 |
| CXCR2 | 1.07279 | 2.013991 | 0.044011 |
| HSD17B6 | 0.840499 | -2.92312 | 0.003465 |
| BARX2 | 1.076335 | 2.14075 | 0.032294 |
| SLC35C1 | 1.280587 | 2.634929 | 0.008415 |
| KCNRG | 1.261339 | 3.246513 | 0.001168 |
| TEX26 | 1.132346 | 2.379645 | 0.017329 |
| TSPAN19 | 1.134585 | 3.003908 | 0.002665 |
| HS3ST1 | 1.240537 | 4.147361 | 3.36E-05 |
| THSD4 | 1.162086 | 2.659856 | 0.007817 |
| DCT | 1.062573 | 2.181455 | 0.02915 |
| ASB4 | 1.071956 | 2.235094 | 0.025411 |
| COL4A1 | 0.902196 | -2.23404 | 0.025481 |
| CCL15 | 1.15708 | 2.173743 | 0.029724 |
| SIAE | 1.239061 | 2.685281 | 0.007247 |
| CAMK2N2 | 0.879943 | -2.53708 | 0.011178 |
| SLC44A3 | 1.156203 | 2.373085 | 0.01764 |
| CAST | 1.26198 | 2.265456 | 0.023485 |
| ATP7B | 1.151678 | 2.260465 | 0.023792 |
| TPT1 | 1.264905 | 2.576176 | 0.00999 |
| LBX2 | 0.887642 | -2.13553 | 0.032718 |
| MGST2 | 1.339691 | 3.356699 | 0.000789 |
| DNAH3 | 1.119819 | 2.042365 | 0.041115 |
| PPARG | 1.098704 | 2.528061 | 0.011469 |
| KLHL41 | 1.032506 | 1.965087 | 0.049404 |
| PLXNA1 | 0.830249 | -2.18836 | 0.028643 |
| WISP1 | 0.899337 | -2.70386 | 0.006854 |
| COL4A2 | 0.900221 | -2.17847 | 0.029371 |
| EHD3 | 1.159642 | 2.203698 | 0.027546 |
| C5orf46 | 1.064921 | 2.14246 | 0.032156 |
| SEMA3G | 0.879432 | -2.26719 | 0.023378 |
| ADAM12 | 0.926017 | -2.18301 | 0.029035 |
| DUSP5 | 1.220728 | 3.162332 | 0.001565 |
| ST3GAL4 | 1.193839 | 2.538301 | 0.011139 |
| GALNT12 | 1.104791 | 2.038623 | 0.041488 |
| KNTC1 | 0.85581 | -1.96613 | 0.049284 |
| ZIC2 | 0.92702 | -2.67581 | 0.007455 |
| APBA2 | 0.867258 | -2.97927 | 0.002889 |
| TTC29 | 1.090258 | 2.178279 | 0.029385 |
| GRB14 | 1.111975 | 3.20027 | 0.001373 |
| GSTA2 | 1.074563 | 2.051559 | 0.040213 |
| DTHD1 | 0.888047 | -2.78995 | 0.005272 |
| PDLIM2 | 1.154832 | 2.212457 | 0.026935 |
| E2F1 | 0.85926 | -2.37443 | 0.017576 |
| COL12A1 | 0.924129 | -1.98098 | 0.047593 |
| SPINK8 | 1.095619 | 2.343038 | 0.019127 |
| ATP2A3 | 0.882351 | -2.2135 | 0.026863 |
| GLP1R | 0.894301 | -2.22958 | 0.025775 |
| ADAMTS12 | 0.931378 | -1.98004 | 0.047699 |
| IL13 | 0.887947 | -2.0706 | 0.038397 |
| CHTF18 | 0.854515 | -2.03673 | 0.041677 |
| RBM24 | 1.058161 | 2.203806 | 0.027538 |
| HAPLN1 | 0.915114 | -2.47231 | 0.013424 |
| EPS8L2 | 1.166849 | 2.001442 | 0.045345 |
| ACSM1 | 1.128519 | 2.352954 | 0.018625 |
| MYL2 | 1.030537 | 2.068711 | 0.038573 |
| ADAMTS2 | 0.907661 | -2.60648 | 0.009148 |
| PHKA1 | 1.1419 | 2.065292 | 0.038895 |
| FMNL3 | 0.749957 | -3.57557 | 0.000349 |
| ONECUT2 | 0.895026 | -3.16824 | 0.001534 |
| BMP8A | 0.914291 | -1.98756 | 0.04686 |
| AQP1 | 0.865813 | -2.05545 | 0.039836 |
| SHOX2 | 0.900719 | -2.85181 | 0.004347 |
| SERPINB1 | 1.127196 | 2.313685 | 0.020685 |
| ZIC5 | 0.93887 | -2.51402 | 0.011936 |
| RAB17 | 1.10892 | 2.72345 | 0.00646 |
| GPR176 | 0.867265 | -3.24105 | 0.001191 |
| C8orf34 | 1.12291 | 2.481191 | 0.013094 |
| C1orf116 | 1.128269 | 2.226159 | 0.026004 |
| SOCS1 | 0.842737 | -3.02347 | 0.002499 |
| ADAMTSL2 | 0.823668 | -3.00195 | 0.002683 |
| SLC15A3 | 0.887533 | -2.23567 | 0.025373 |
| CCDC14 | 0.797633 | -2.54825 | 0.010827 |
| CDX1 | 0.878104 | -2.79915 | 0.005124 |
| ELOVL6 | 1.284086 | 4.103455 | 4.07E-05 |
| BTC | 1.119625 | 3.115074 | 0.001839 |
| TCF24 | 0.885172 | -2.53674 | 0.011189 |
| CHST2 | 0.906182 | -2.39237 | 0.01674 |
| TLX2 | 0.909327 | -2.65225 | 0.007996 |
| ZAN | 0.903217 | -3.18508 | 0.001447 |
| MYL1 | 1.02747 | 1.99227 | 0.046341 |
| SH2D4B | 0.906762 | -1.96147 | 0.049824 |
| SAA2-SAA4 | 1.064861 | 2.210837 | 0.027047 |
| TNFSF4 | 0.910523 | -2.01168 | 0.044254 |
| DDX11 | 0.817871 | -2.66414 | 0.007719 |
| CELSR3 | 0.819737 | -4.48179 | 7.40E-06 |
| GPC2 | 0.886711 | -2.70862 | 0.006756 |
| CAVIN2 | 1.096832 | 2.003077 | 0.045169 |
| CHN1 | 0.851631 | -2.66085 | 0.007794 |
| TMEM26 | 0.908972 | -2.06834 | 0.038608 |
| ADAMTS14 | 0.88918 | -2.30278 | 0.021291 |
| TERT | 0.916979 | -2.23845 | 0.025192 |
| RNASE10 | 0.929689 | -2.82394 | 0.004744 |
| HSPB7 | 1.04145 | 1.984408 | 0.04721 |
| RSPO1 | 0.919043 | -2.19144 | 0.02842 |
| BRINP1 | 1.100233 | 2.703672 | 0.006858 |
| ZNF469 | 0.916935 | -2.11456 | 0.034468 |
| COL16A1 | 0.888144 | -2.20604 | 0.027381 |
| SALL4 | 0.92262 | -2.10497 | 0.035294 |
| PRPH2 | 0.902785 | -2.13704 | 0.032595 |
| TTC16 | 0.902712 | -1.98558 | 0.04708 |
| ONECUT3 | 0.881427 | -3.26884 | 0.00108 |
| ADCY2 | 1.072473 | 2.513074 | 0.011968 |
| CTSG | 0.92479 | -2.1143 | 0.034489 |
| CSMD2 | 0.913162 | -2.2638 | 0.023586 |
| BEND7 | 1.090864 | 2.183279 | 0.029015 |
| LAIR2 | 0.901086 | -2.48975 | 0.012783 |
| NOX4 | 0.898486 | -2.24255 | 0.024926 |
| SLC17A9 | 0.868858 | -2.90219 | 0.003706 |
| GFI1 | 0.870149 | -2.54312 | 0.010987 |
| FBXO43 | 0.89389 | -2.33344 | 0.019625 |
| AADAC | 1.05271 | 2.075946 | 0.037899 |
| ANKRD53 | 0.872388 | -2.07104 | 0.038355 |
| COL6A3 | 0.914483 | -2.37467 | 0.017565 |
| IRF9 | 0.87443 | -1.98084 | 0.047609 |
| FADD | 1.124008 | 2.080655 | 0.037465 |
| TNFRSF9 | 0.896191 | -2.39202 | 0.016756 |
| FBXO41 | 0.877785 | -2.22754 | 0.025911 |
| IER5L | 0.854523 | -2.25244 | 0.024294 |
| COL6A1 | 0.89968 | -2.29375 | 0.021805 |
| NKD2 | 0.876405 | -2.79238 | 0.005232 |
| MYO7A | 0.86488 | -2.25508 | 0.024129 |
| TNFRSF4 | 0.803943 | -4.30731 | 1.65E-05 |
| SMC1B | 0.937034 | -2.13515 | 0.032748 |
| PTX3 | 1.090565 | 2.75394 | 0.005888 |
| CEBPE | 0.863607 | -3.44225 | 0.000577 |
| GRK7 | 0.876839 | -2.53079 | 0.011381 |
| SLC6A7 | 0.826696 | -3.47319 | 0.000514 |
| CXCL13 | 0.938295 | -2.16668 | 0.030259 |
| KLHDC7B | 0.925747 | -2.4332 | 0.014966 |
| SYNGR3 | 0.886334 | -2.81604 | 0.004862 |
| ALG1L | 0.904344 | -2.16265 | 0.030568 |
| DES | 1.032346 | 2.030673 | 0.042288 |
| NIPAL1 | 1.117214 | 1.963238 | 0.049619 |
| LHX9 | 0.939073 | -2.02885 | 0.042474 |
| SGIP1 | 0.872907 | -2.61572 | 0.008904 |
| MSS51 | 0.807517 | -2.90022 | 0.003729 |
| F2RL2 | 0.909159 | -2.52801 | 0.011471 |
| TTLL9 | 0.86296 | -2.31763 | 0.020469 |
| C9orf66 | 1.112137 | 2.012936 | 0.044121 |
| CACNA1B | 0.938888 | -2.42143 | 0.01546 |
| AMZ1 | 0.887585 | -2.35976 | 0.018287 |
| NETO1 | 0.910703 | -2.41105 | 0.015907 |
| TTBK1 | 0.794183 | -4.318 | 1.57E-05 |
| ASPN | 0.906947 | -2.6192 | 0.008814 |
| ONECUT1 | 0.905639 | -2.07502 | 0.037985 |
| MPZL3 | 1.143644 | 2.102251 | 0.035531 |
| CTLA4 | 0.873866 | -3.20716 | 0.001341 |
| CRTAC1 | 1.066236 | 2.145809 | 0.031888 |
| GPR161 | 0.845114 | -2.56168 | 0.010417 |
| MOV10L1 | 0.872344 | -2.80084 | 0.005097 |
| CLDN14 | 0.908312 | -2.15707 | 0.031 |
| ITGA1 | 0.816388 | -3.22698 | 0.001251 |
| ADGRD2 | 0.870568 | -3.45616 | 0.000548 |
| ANGPT2 | 0.880164 | -2.07622 | 0.037874 |
| ABCB4 | 0.842957 | -2.80593 | 0.005017 |
| CCDC113 | 1.202073 | 3.130892 | 0.001743 |
| AVPR2 | 0.835572 | -3.51084 | 0.000447 |
| SLC5A9 | 0.875517 | -2.41309 | 0.015818 |
| CACNB2 | 0.834497 | -2.45549 | 0.014069 |
| AC024940.1 | 0.923371 | -2.41748 | 0.015628 |
| C20orf204 | 0.885348 | -2.2789 | 0.022673 |
| COL8A1 | 0.926917 | -2.0292 | 0.042438 |
| MYF6 | 1.03773 | 2.077224 | 0.037781 |
| TMC8 | 0.754814 | -4.41534 | 1.01E-05 |
| USP2 | 1.107883 | 2.684681 | 0.00726 |
| IFI30 | 0.865823 | -2.08602 | 0.036977 |
| PBX4 | 0.812357 | -3.21628 | 0.001299 |
| CLEC12A | 0.903919 | -2.37775 | 0.017419 |
| SIGLEC12 | 0.929031 | -2.12577 | 0.033522 |
| SMPX | 1.041659 | 2.336171 | 0.019482 |
| PLA2G4C | 0.863012 | -2.52631 | 0.011527 |
| KRT39 | 1.213859 | 4.646285 | 3.38E-06 |
| PRSS53 | 0.894034 | -2.04972 | 0.040391 |
| FCHO1 | 0.898109 | -1.96344 | 0.049595 |
| LAG3 | 0.909734 | -2.16917 | 0.03007 |
| FOXP3 | 0.877717 | -2.72414 | 0.006447 |
| AC040162.1 | 0.804917 | -2.81708 | 0.004846 |
| CNGA1 | 1.108046 | 2.006994 | 0.04475 |
| C19orf84 | 0.910155 | -2.23504 | 0.025415 |
| EPHA2 | 1.158377 | 2.174669 | 0.029655 |
| FMN1 | 1.138665 | 2.121924 | 0.033844 |
| ALKBH6 | 0.822925 | -2.50596 | 0.012212 |
| TMEM150B | 0.890947 | -2.35799 | 0.018374 |
| NCALD | 0.871809 | -2.53737 | 0.011169 |
| DNER | 1.062363 | 1.968434 | 0.049018 |
| PGGHG | 0.878566 | -2.20232 | 0.027643 |
| ZBTB32 | 0.842982 | -3.3175 | 0.000908 |
| HENMT1 | 0.835358 | -3.05584 | 0.002244 |
| AC005726.2 | 0.839672 | -2.09131 | 0.0365 |
| PDE4B | 0.885834 | -2.21915 | 0.026476 |
| LIMD2 | 0.746518 | -4.24025 | 2.23E-05 |
| CHRNA6 | 0.863913 | -3.05718 | 0.002234 |
| P2RX6 | 1.100066 | 2.311702 | 0.020794 |
| SLAMF8 | 0.885373 | -2.29386 | 0.021798 |
| TMEM262 | 0.788722 | -2.93824 | 0.003301 |
| RXFP3 | 0.876639 | -2.04072 | 0.041278 |
| BLACE | 0.833092 | -2.54233 | 0.011012 |
| RASL12 | 0.85464 | -2.32669 | 0.019982 |
| RTEL1-TNFRSF6B | 0.815724 | -2.67889 | 0.007387 |
| KCNAB3 | 0.813504 | -3.2762 | 0.001052 |
| SLC26A9 | 0.933636 | -2.30048 | 0.021421 |
| SYCP2 | 0.896156 | -3.13706 | 0.001706 |
| IL4I1 | 0.896676 | -2.35596 | 0.018475 |
| CEND1 | 0.879902 | -2.31759 | 0.020472 |
| ZFP42 | 1.060166 | 2.178358 | 0.029379 |
| RXFP1 | 0.90191 | -2.24537 | 0.024745 |
| SLC14A2 | 0.872207 | -2.34363 | 0.019097 |
| AC135178.2 | 0.885938 | -1.98112 | 0.047578 |
| EPYC | 0.932928 | -2.24479 | 0.024781 |
| PAG1 | 0.840184 | -2.60269 | 0.00925 |
| ADAM33 | 0.885663 | -2.48 | 0.013138 |
| CCL5 | 0.898657 | -2.44352 | 0.014545 |
| GNG8 | 0.867604 | -2.99259 | 0.002766 |
| CLEC1B | 0.900823 | -2.14357 | 0.032067 |
| ANKLE1 | 0.879815 | -2.73372 | 0.006262 |
| NKX6-2 | 1.165794 | 2.27816 | 0.022717 |
| FOXD4L1 | 0.814594 | -3.18191 | 0.001463 |
| AMHR2 | 0.898092 | -2.07554 | 0.037937 |
| ERVMER34-1 | 1.148615 | 2.725787 | 0.006415 |
| PCDH17 | 0.875277 | -2.58004 | 0.009879 |
| COL6A2 | 0.902141 | -2.21363 | 0.026854 |
| NTRK1 | 0.81135 | -3.32953 | 0.00087 |
| IL2RA | 0.879429 | -2.67395 | 0.007496 |
| RUNX3 | 0.885767 | -2.0505 | 0.040315 |
| CMTM1 | 0.849497 | -2.77992 | 0.005437 |
| ITGA2B | 0.852526 | -2.67104 | 0.007562 |
| NAT8L | 1.07957 | 2.165304 | 0.030364 |
| ITGAX | 0.868048 | -2.53953 | 0.0111 |
| ADCY10 | 0.909964 | -2.05905 | 0.039489 |
| DOK3 | 0.80776 | -3.1348 | 0.00172 |
| PCDH20 | 1.156277 | 2.260278 | 0.023804 |
| TNFRSF25 | 0.856928 | -2.54435 | 0.010948 |
| PGD | 1.17881 | 2.635104 | 0.008411 |
| STRA6 | 0.928258 | -2.11573 | 0.034368 |
| MSH5 | 0.874411 | -2.25969 | 0.023841 |
| TIGIT | 0.869874 | -3.27872 | 0.001043 |
| REEP6 | 1.17116 | 2.869132 | 0.004116 |
| RYR3 | 0.873608 | -2.86356 | 0.004189 |
| ZBP1 | 0.877823 | -3.33306 | 0.000859 |
| GRIA3 | 0.885314 | -3.06526 | 0.002175 |
| MMP19 | 0.864263 | -2.7059 | 0.006812 |
| CYSLTR2 | 0.896471 | -2.20115 | 0.027725 |
| TNFRSF18 | 0.872382 | -2.97249 | 0.002954 |
| MAST1 | 0.909446 | -2.02584 | 0.042781 |
| FOXS1 | 0.903007 | -2.0428 | 0.041072 |
| PAPLN | 0.84919 | -2.75531 | 0.005864 |
| TRIML2 | 1.052074 | 2.1143 | 0.03449 |
| KLHL14 | 0.910084 | -2.09662 | 0.036027 |
| SP9 | 0.888959 | -3.54507 | 0.000393 |
| CCR8 | 0.92242 | -2.21712 | 0.026615 |
| ABCA6 | 0.886722 | -2.30879 | 0.020955 |
| FADS2 | 0.884191 | -2.745 | 0.006051 |
| SYT14 | 1.071278 | 2.14632 | 0.031847 |
| LARGE2 | 1.176571 | 2.647502 | 0.008109 |
| POU5F1 | 0.852665 | -2.73147 | 0.006305 |
| C10orf35 | 1.132685 | 1.986176 | 0.047014 |
| GPR150 | 0.815938 | -3.49551 | 0.000473 |
| AC020922.1 | 0.893214 | -2.43329 | 0.014962 |
| HMSD | 0.91397 | -2.22663 | 0.025972 |
| IL12RB2 | 0.931858 | -2.00587 | 0.04487 |
| BHMG1 | 0.892401 | -2.14066 | 0.032302 |
| ZFR2 | 0.909972 | -2.92119 | 0.003487 |
| PILRA | 0.876436 | -2.36313 | 0.018121 |
| CHIT1 | 0.939529 | -2.54788 | 0.010838 |
| CCL11 | 0.90873 | -3.0052 | 0.002654 |
| ZP3 | 1.155361 | 1.983112 | 0.047355 |
| GNLY | 0.928355 | -1.97546 | 0.048216 |
| HMX2 | 0.912694 | -2.5386 | 0.01113 |
| MYO1G | 0.834574 | -3.1501 | 0.001632 |
| GTSF1L | 0.819956 | -3.27751 | 0.001047 |
| ICOS | 0.883296 | -2.94414 | 0.003239 |
| FOXRED2 | 0.835245 | -2.60975 | 0.009061 |
| OLFML2B | 0.898257 | -2.18206 | 0.029105 |
| SEC61G | 1.139721 | 2.143648 | 0.032061 |
| SHISA7 | 0.914524 | -2.02384 | 0.042987 |
| C17orf99 | 0.890959 | -2.29431 | 0.021773 |
| CLDN8 | 1.05108 | 2.378223 | 0.017396 |
| SP140 | 0.877445 | -2.75179 | 0.005927 |
| PTX4 | 0.869378 | -3.30342 | 0.000955 |
| ZNF541 | 0.881223 | -3.89001 | 0.0001 |
| ASNS | 1.163316 | 2.024077 | 0.042962 |
| IL21R | 0.868327 | -3.24334 | 0.001181 |
| POU2F2 | 0.869975 | -2.2538 | 0.024209 |
| ACAN | 0.921872 | -2.0718 | 0.038284 |
| TMEM130 | 0.887232 | -2.75251 | 0.005914 |
| SLC22A11 | 0.880648 | -2.43506 | 0.014889 |
| MIXL1 | 0.917861 | -2.0964 | 0.036046 |
| SPDYA | 0.819874 | -3.00734 | 0.002635 |
| PDGFRB | 0.884959 | -2.49902 | 0.012454 |
| FRMD5 | 1.083793 | 2.008565 | 0.044583 |
| F5 | 0.895627 | -2.52318 | 0.01163 |
| MT1X | 1.182192 | 3.034062 | 0.002413 |
| ITGA11 | 0.921703 | -2.16716 | 0.030222 |
| YJEFN3 | 0.902629 | -2.17906 | 0.029327 |
| AKAP5 | 0.830584 | -3.20569 | 0.001347 |
| PSTPIP1 | 0.841906 | -2.71668 | 0.006594 |
| EPO | 0.924207 | -2.1499 | 0.031563 |
| TMC4 | 1.090924 | 1.966863 | 0.049199 |
| AZIN2 | 0.865149 | -2.25109 | 0.02438 |
| TRAF1 | 0.804998 | -3.15687 | 0.001595 |
| ABCD2 | 0.898788 | -2.70427 | 0.006845 |
| OR51E1 | 0.893919 | -1.97201 | 0.048608 |
| STAG3 | 0.878435 | -3.44743 | 0.000566 |
| C1orf186 | 0.891371 | -3.43198 | 0.000599 |
| MEI1 | 0.875239 | -3.28262 | 0.001028 |
| SPAG4 | 0.875006 | -2.09953 | 0.03577 |
| ZC3H12D | 0.81567 | -3.88099 | 0.000104 |
| SLFN12L | 0.844522 | -2.76062 | 0.005769 |
| MS4A14 | 0.89229 | -2.12478 | 0.033605 |
| TNFRSF8 | 0.869398 | -2.75377 | 0.005891 |
| LYG1 | 0.848939 | -3.05122 | 0.002279 |
| TINAG | 0.872029 | -2.41197 | 0.015867 |
| LAT | 0.823669 | -3.57137 | 0.000355 |
| SLC35G5 | 0.833054 | -3.44225 | 0.000577 |
| HTR3E | 0.854714 | -3.06156 | 0.002202 |
| ARHGAP4 | 0.805021 | -3.95239 | 7.74E-05 |
| TMEM200C | 0.924905 | -2.12572 | 0.033527 |
| PLS1 | 1.126981 | 2.462473 | 0.013798 |
| ACOD1 | 0.91379 | -2.04541 | 0.040814 |
| KLHL35 | 0.920121 | -2.0319 | 0.042164 |
| CPAMD8 | 0.874634 | -2.26962 | 0.02323 |
| RP1 | 1.107568 | 2.460346 | 0.01388 |
| KCNF1 | 1.110234 | 2.616454 | 0.008885 |
| GUCY1B3 | 0.855417 | -2.85826 | 0.00426 |
| SCNN1D | 0.913016 | -2.28757 | 0.022163 |
| RHOF | 0.836728 | -2.84061 | 0.004503 |
| TRPC6 | 0.851478 | -2.40664 | 0.0161 |
| TFPI2 | 1.060164 | 1.975041 | 0.048263 |
| TBR1 | 0.895909 | -1.97468 | 0.048305 |
| UNC13A | 0.903666 | -2.0118 | 0.044241 |
| ANKRD1 | 1.040693 | 2.156056 | 0.031079 |
| AC091980.2 | 0.83415 | -2.6757 | 0.007457 |
| INSM1 | 0.902596 | -2.46467 | 0.013714 |
| TMEM235 | 0.854962 | -2.59315 | 0.00951 |
| TDRD6 | 0.889769 | -2.00872 | 0.044566 |
| AC092821.1 | 0.79893 | -4.05587 | 4.99E-05 |
| TREML2 | 0.872474 | -2.81443 | 0.004886 |
| KCNJ10 | 0.868616 | -3.44621 | 0.000569 |
| TMEM114 | 0.879244 | -2.40645 | 0.016109 |
| LINC00282 | 0.793253 | -3.05538 | 0.002248 |
| CCDC155 | 0.906871 | -2.50685 | 0.012181 |
| POU4F1 | 0.941524 | -2.07917 | 0.037602 |
| DNAJB13 | 0.894887 | -2.33843 | 0.019365 |
| PYGL | 1.170194 | 2.83674 | 0.004558 |
| HNF1A | 0.854868 | -2.86239 | 0.004205 |
| CLEC6A | 0.869277 | -3.02839 | 0.002459 |
| HIST1H3F | 1.150047 | 2.348667 | 0.018841 |
| SDK1 | 0.899794 | -2.26636 | 0.02343 |
| FNDC1 | 0.933917 | -2.16501 | 0.030387 |
| A4GNT | 0.864148 | -2.19913 | 0.027869 |
| GRIP2 | 0.900702 | -2.20408 | 0.027519 |
| AF130351.1 | 0.821111 | -2.64908 | 0.008071 |
| TMEM155 | 0.847127 | -3.03897 | 0.002374 |
| SOX1 | 1.057243 | 2.156163 | 0.031071 |
| PIEZO2 | 0.898613 | -2.34065 | 0.01925 |
| FAM159A | 0.88611 | -2.51963 | 0.011748 |
| JCHAIN | 0.917766 | -3.42743 | 0.000609 |
| APOBEC3H | 0.895211 | -2.19795 | 0.027953 |
| PTPN7 | 0.844336 | -3.3533 | 0.000799 |
| NKX3-2 | 0.913837 | -2.50783 | 0.012147 |
| SLC28A3 | 0.916308 | -2.07023 | 0.038431 |
| NCR1 | 0.854639 | -3.3509 | 0.000805 |
| PRF1 | 0.883467 | -2.6041 | 0.009211 |
| RAB39A | 0.884399 | -2.69755 | 0.006985 |
| ETV3L | 0.875651 | -2.62159 | 0.008752 |
| MT4 | 1.08692 | 2.119692 | 0.034032 |
| TLE6 | 1.112444 | 2.144102 | 0.032025 |
| IL12RB1 | 0.875131 | -2.74245 | 0.006098 |
| PCOLCE2 | 1.075319 | 2.384341 | 0.01711 |
| HTR1F | 0.843639 | -3.08032 | 0.002068 |
| BEST2 | 0.917387 | -2.60908 | 0.009079 |
| SSTR2 | 0.869244 | -2.74446 | 0.006061 |
| BRICD5 | 0.882884 | -2.30763 | 0.02102 |
| PITX2 | 1.064769 | 2.169157 | 0.030071 |
| TIGD3 | 0.876124 | -2.03681 | 0.041669 |
| DKK1 | 1.115868 | 3.754813 | 0.000173 |
| A1BG | 0.868773 | -2.29629 | 0.021659 |
| RIPOR3 | 0.833802 | -3.85543 | 0.000116 |
| TFEC | 0.903519 | -2.11601 | 0.034344 |
| FAM221B | 0.895499 | -1.9778 | 0.047952 |
| FCRL3 | 0.897388 | -3.24558 | 0.001172 |
| LSAMP | 0.90179 | -2.46949 | 0.013531 |
| GDPD4 | 0.889441 | -2.00239 | 0.045243 |
| SYTL5 | 1.078006 | 2.380663 | 0.017282 |
| INSM2 | 0.840202 | -2.14944 | 0.031599 |
| TEX22 | 0.810396 | -3.4423 | 0.000577 |
| INSRR | 0.883992 | -2.52912 | 0.011435 |
| CLEC17A | 0.882726 | -3.27484 | 0.001057 |
| KLRF1 | 0.908072 | -2.07093 | 0.038365 |
| GNRH1 | 0.852336 | -2.91094 | 0.003603 |
| TMEM151B | 0.905158 | -2.71376 | 0.006652 |
| SHCBP1L | 0.897889 | -2.30104 | 0.021389 |
| PXYLP1 | 0.87713 | -2.32632 | 0.020001 |
| C4orf50 | 0.886104 | -2.15739 | 0.030976 |
| TREML1 | 0.804521 | -3.54671 | 0.00039 |
| EML5 | 0.86628 | -3.89628 | 9.77E-05 |
| CASQ2 | 1.044369 | 2.102384 | 0.03552 |
| CARMIL2 | 0.810099 | -3.71423 | 0.000204 |
| GOLGA8A | 0.919209 | -2.01097 | 0.044328 |
| SERPINA9 | 0.921447 | -2.56603 | 0.010287 |
| CTTN | 1.171697 | 2.753241 | 0.005901 |
| HAVCR1 | 0.851419 | -2.53234 | 0.01133 |
| RNFT2 | 0.917608 | -1.97374 | 0.048412 |
| EOMES | 0.879054 | -2.96973 | 0.002981 |
| SIRPG | 0.884002 | -2.93876 | 0.003295 |
| CSH2 | 0.791203 | -2.24134 | 0.025004 |
| SPIB | 0.912701 | -2.82848 | 0.004677 |
| CAMKV | 0.845372 | -3.15954 | 0.00158 |
| COL8A2 | 0.84011 | -3.21112 | 0.001322 |
| ITGAM | 0.87648 | -2.58409 | 0.009764 |
| RHBDL3 | 0.920512 | -2.08107 | 0.037427 |
| IL22RA2 | 0.916504 | -2.35646 | 0.01845 |
| SLC5A2 | 0.849529 | -2.70673 | 0.006795 |
| WNT7A | 1.0788 | 2.582247 | 0.009816 |
| SPATC1 | 0.815422 | -3.59206 | 0.000328 |
| TRPC3 | 0.832035 | -3.10591 | 0.001897 |
| DKKL1 | 0.891656 | -2.51419 | 0.011931 |
| ACPT | 0.889386 | -2.00746 | 0.044701 |
| CCL26 | 1.079121 | 2.120758 | 0.033942 |
| TSGA10IP | 0.889298 | -2.63035 | 0.00853 |
| GZMB | 0.919601 | -2.13158 | 0.033041 |
| CCNA1 | 1.064919 | 2.578872 | 0.009912 |
| ZGLP1 | 0.847052 | -2.6707 | 0.007569 |
| SNAP25 | 0.880799 | -2.82161 | 0.004778 |
| VWCE | 0.864751 | -2.52731 | 0.011494 |
| AC107871.1 | 1.160604 | 2.451199 | 0.014238 |
| NANOS3 | 0.805261 | -3.36814 | 0.000757 |
| WDR97 | 0.856721 | -3.53591 | 0.000406 |
| PRRT2 | 0.857738 | -2.83714 | 0.004552 |
| SSTR3 | 0.8836 | -2.91938 | 0.003507 |
| ANKFN1 | 1.079725 | 2.169927 | 0.030012 |
| CHRNB4 | 1.082369 | 2.40485 | 0.016179 |
| TMPRSS9 | 0.881877 | -2.19755 | 0.027981 |
| PROX2 | 0.875151 | -2.39727 | 0.016518 |
| OR7D4 | 0.550282 | -1.98824 | 0.046785 |
| GABRR2 | 0.846581 | -2.78621 | 0.005333 |
| ZPBP2 | 0.88439 | -2.43605 | 0.014849 |
| C8G | 0.895403 | -2.24984 | 0.024459 |
| TMEM105 | 1.110352 | 2.080032 | 0.037523 |
| TMEM89 | 0.825685 | -2.49667 | 0.012537 |
| CLEC4E | 0.903572 | -2.32777 | 0.019925 |
| SLFN14 | 0.820948 | -2.66468 | 0.007706 |
| TRIM67 | 0.886129 | -2.15129 | 0.031453 |
| SPINK6 | 0.950512 | -2.5271 | 0.011501 |
| TMEM156 | 0.903163 | -2.08539 | 0.037034 |
| AMER2 | 0.860154 | -2.56533 | 0.010308 |
| AL603832.3 | 0.830221 | -2.63825 | 0.008334 |
| TBC1D26 | 0.820622 | -2.09916 | 0.035803 |
| TRIM73 | 0.88082 | -2.21273 | 0.026917 |
| AC009336.2 | 0.766025 | -3.92969 | 8.51E-05 |
| SPOCK1 | 1.079066 | 2.177876 | 0.029415 |
| IL27 | 0.891704 | -2.21781 | 0.026568 |
| ITGAD | 0.865492 | -2.89772 | 0.003759 |
| MUM1L1 | 1.089473 | 3.290247 | 0.001001 |
| RSPH6A | 0.869231 | -2.60725 | 0.009127 |
| RBPJL | 0.888083 | -2.10442 | 0.035342 |
| ADRB3 | 0.886311 | -2.04118 | 0.041233 |
| CD40LG | 0.914876 | -2.29023 | 0.022008 |
| SPDYE2 | 0.861722 | -2.54554 | 0.010911 |
| CPXM1 | 0.917793 | -2.05366 | 0.040008 |
| C6orf223 | 0.919832 | -2.50311 | 0.012311 |
| AFF3 | 0.891803 | -2.82713 | 0.004697 |
| ACOXL | 0.844543 | -3.11721 | 0.001826 |
| ZCCHC18 | 0.869625 | -2.60604 | 0.00916 |
| CD38 | 0.900058 | -2.58686 | 0.009685 |
| ELOVL2 | 0.8926 | -2.35119 | 0.018714 |
| CDH18 | 0.899059 | -2.40024 | 0.016384 |
| CCL25 | 0.878346 | -2.41873 | 0.015575 |
| KCNS1 | 0.950423 | -2.08969 | 0.036645 |
| UGT1A8 | 1.047679 | 1.976034 | 0.048151 |
| PHACTR3 | 0.911934 | -2.25759 | 0.023971 |
| SPSB4 | 0.933258 | -2.01332 | 0.044081 |
| DEFA3 | 1.152591 | 2.177293 | 0.029459 |
| JAKMIP1 | 0.900371 | -2.45416 | 0.014121 |
| HCAR1 | 0.907865 | -2.62151 | 0.008754 |
| CTSE | 0.924972 | -2.07666 | 0.037833 |
| SLC2A7 | 0.878367 | -1.97768 | 0.047964 |
| TPTE | 0.806525 | -2.20219 | 0.027652 |
| FAM71E2 | 0.846196 | -1.9617 | 0.049798 |
| LRRC74A | 0.854647 | -2.20084 | 0.027747 |
| KIR3DX1 | 0.797425 | -4.01537 | 5.94E-05 |
| COLEC11 | 0.902091 | -2.56514 | 0.010314 |
| MAJIN | 0.920215 | -2.02969 | 0.042388 |
| CCBE1 | 1.095763 | 2.567822 | 0.010234 |
| DRGX | 0.888344 | -2.41977 | 0.01553 |
| FAM196A | 0.845942 | -2.66878 | 0.007613 |
| KLRC2 | 0.905679 | -2.18371 | 0.028983 |
| DPEP1 | 0.922074 | -2.13244 | 0.032971 |
| SLC16A8 | 0.913854 | -1.97846 | 0.047877 |
| AIPL1 | 0.857314 | -2.99154 | 0.002776 |
| MUC17 | 0.870348 | -2.59579 | 0.009437 |
| NKX2-3 | 0.935541 | -2.55833 | 0.010518 |
| CRYGD | 0.515822 | -2.14457 | 0.031987 |
| CD27 | 0.849682 | -3.86499 | 0.000111 |
| IGDCC3 | 0.850234 | -2.57935 | 0.009899 |
| ENTHD1 | 0.902964 | -2.0918 | 0.036456 |
| GPR141 | 0.900011 | -2.32674 | 0.019979 |
| ZNF80 | 0.864772 | -2.92227 | 0.003475 |
| TTC24 | 0.848686 | -3.78175 | 0.000156 |
| ZNF560 | 1.123988 | 2.160598 | 0.030726 |
| NPHS1 | 0.917451 | -2.0319 | 0.042164 |
| IL21 | 0.813677 | -2.73918 | 0.006159 |
| RNF212 | 0.935894 | -2.22914 | 0.025805 |
| ARHGEF33 | 0.893207 | -2.51838 | 0.01179 |
| PAK3 | 0.922024 | -2.15643 | 0.03105 |
| KLRK1 | 0.821939 | -4.10404 | 4.06E-05 |
| GAD2 | 0.859857 | -2.01633 | 0.043766 |
| AICDA | 0.846455 | -3.27376 | 0.001061 |
| FCRLA | 0.879089 | -3.54929 | 0.000386 |
| PRND | 0.914377 | -2.49176 | 0.012711 |
| STAR | 0.895243 | -2.65253 | 0.007989 |
| TCP11 | 0.894343 | -3.51238 | 0.000444 |
| MESP2 | 0.897577 | -2.79269 | 0.005227 |
| GBP7 | 0.848599 | -2.90713 | 0.003648 |
| SLC35G3 | 0.880407 | -2.77567 | 0.005509 |
| VCAM1 | 0.892092 | -2.95036 | 0.003174 |
| CYP4F11 | 1.057117 | 2.065547 | 0.038871 |
| CD79A | 0.885769 | -4.04449 | 5.24E-05 |
| HOXA6 | 0.912779 | -2.47439 | 0.013346 |
| IMPG2 | 0.835865 | -2.79767 | 0.005147 |
| LYPD4 | 0.85634 | -2.54852 | 0.010818 |
| NANOS2 | 0.797779 | -2.66112 | 0.007788 |
| CD19 | 0.871773 | -3.98303 | 6.80E-05 |
| SERTM1 | 1.112395 | 2.358104 | 0.018369 |
| CAPN8 | 1.082968 | 2.227952 | 0.025884 |
| PLA2G2D | 0.929226 | -2.73059 | 0.006322 |
| LAX1 | 0.863098 | -3.65236 | 0.00026 |
| DNMT3L | 0.824394 | -2.37067 | 0.017756 |
| HORMAD1 | 0.959043 | -2.10668 | 0.035146 |
| DMRTC2 | 0.860631 | -2.40402 | 0.016216 |
| FAM205A | 0.880959 | -2.58391 | 0.009769 |
| DCC | 0.922435 | -1.9827 | 0.047401 |
| HEATR9 | 0.868171 | -2.49527 | 0.012586 |
| FOXA2 | 1.078676 | 2.706964 | 0.00679 |
| BTN1A1 | 0.893816 | -1.99475 | 0.04607 |
| FCRL5 | 0.896913 | -3.95142 | 7.77E-05 |
| CRX | 0.852096 | -2.48062 | 0.013115 |
| C1orf167 | 0.851352 | -2.71931 | 0.006542 |
| IDO2 | 0.859801 | -3.22522 | 0.001259 |
| NPS | 1.191502 | 2.397776 | 0.016495 |
| NANOG | 0.824056 | -2.43862 | 0.014743 |
| BLK | 0.892762 | -3.48947 | 0.000484 |
| SFTPA2 | 1.068714 | 2.274233 | 0.022952 |
| IGLL1 | 0.873052 | -2.93069 | 0.003382 |
| ZYG11A | 0.91697 | -2.60973 | 0.009061 |
| NTN3 | 0.850829 | -3.09946 | 0.001939 |
| TSPAN16 | 0.805511 | -2.81053 | 0.004946 |
| OR5M11 | 1.17012 | 2.315678 | 0.020576 |
| CACNG2 | 0.85922 | -2.08773 | 0.036822 |
| LHX2 | 0.940293 | -2.06396 | 0.039022 |
| PGLYRP2 | 0.897899 | -2.60433 | 0.009205 |
| ASCL4 | 0.895238 | -2.30638 | 0.021089 |
| ZSCAN10 | 0.864694 | -2.06284 | 0.039128 |
| RTL1 | 1.148743 | 3.219144 | 0.001286 |
| CREB3L3 | 0.847235 | -2.94014 | 0.003281 |
| MS4A12 | 0.733747 | -2.08021 | 0.037506 |
| NR0B1 | 1.102539 | 3.067124 | 0.002161 |
| CHGB | 1.061235 | 2.762391 | 0.005738 |
| TMSB4Y | 0.920024 | -2.87439 | 0.004048 |
| AC136428.1 | 0.902824 | -2.91096 | 0.003603 |
| IGLL5 | 0.925926 | -3.48256 | 0.000497 |
| LRRC9 | 1.102803 | 2.108772 | 0.034964 |
| PNMA3 | 0.914753 | -2.26204 | 0.023695 |
| IL17REL | 0.871467 | -3.57704 | 0.000348 |
| FCRL2 | 0.874613 | -3.74629 | 0.000179 |
| UGT2B17 | 0.921242 | -2.32334 | 0.020161 |
| ECEL1 | 0.893348 | -2.94193 | 0.003262 |
| UGT2A1 | 1.078302 | 2.157648 | 0.030955 |
| DEFB118 | 0.800046 | -1.97221 | 0.048586 |
| KIF25 | 0.898919 | -2.19208 | 0.028374 |
| DDX43 | 0.913203 | -2.50535 | 0.012233 |
| GPHA2 | 0.863625 | -2.60812 | 0.009104 |
| SLC22A6 | 0.847607 | -2.20919 | 0.027161 |
| FCRL1 | 0.848483 | -4.13141 | 3.61E-05 |
| H1FNT | 0.83301 | -2.74792 | 0.005998 |
| SEMG1 | 1.114125 | 2.103448 | 0.035427 |
| MS4A1 | 0.898966 | -3.88348 | 0.000103 |
| FCRL4 | 0.850398 | -3.66082 | 0.000251 |
| KRT20 | 1.10733 | 3.148498 | 0.001641 |
| PNLDC1 | 0.942867 | -2.20786 | 0.027254 |
| KCNJ18 | 0.92401 | -2.69664 | 0.007004 |
| UGT1A4 | 0.857022 | -2.00783 | 0.044661 |
| LGR5 | 0.928462 | -2.5798 | 0.009886 |
| GRIN2A | 0.926399 | -2.59194 | 0.009544 |
| CBLN2 | 0.882099 | -3.88519 | 0.000102 |
| VRTN | 0.85898 | -2.1123 | 0.03466 |
| CXCR5 | 0.850892 | -3.53293 | 0.000411 |
| KIR2DL1 | 0.857127 | -2.44691 | 0.014409 |
| SP7 | 0.883678 | -2.229 | 0.025814 |
| DEFB124 | 0.870775 | -2.42956 | 0.015117 |
| RHOXF1 | 0.863205 | -2.72828 | 0.006367 |
| PRSS57 | 0.802403 | -2.95257 | 0.003151 |
| CATSPER4 | 0.846423 | -2.56156 | 0.01042 |
| GBX1 | 0.892396 | -2.42723 | 0.015215 |
| WIF1 | 1.041761 | 2.010083 | 0.044422 |
| AIRE | 0.886353 | -2.21966 | 0.026442 |
| OR5C1 | 0.805793 | -2.19558 | 0.028122 |
| PRR23C | 0.804897 | -2.00643 | 0.04481 |
| LELP1 | 0.824893 | -2.20441 | 0.027495 |
